# Hydrogel Fiber Evaporator with Vertical Channels Integrated with Dual Heat Supply/Insulation Model for Continuous Solar Desalination

**DOI:** 10.1007/s40820-026-02120-z

**Published:** 2026-02-28

**Authors:** Tian Wang, Shuai Gao, Yongli Yu, Zhigang Chen, Lili Wang, Xiansheng Zhang

**Affiliations:** 1 Shandong Key Laboratory of Medical and Health Textile MaterialsCollege of Textiles and ClothingState Key Laboratory of Bio-Fibers and Eco-Textiles, Qingdao University, Qingdao, 266071 People’s Republic of China; 2 State Key Laboratory of Advanced Fiber Materials, College of Materials Science and Engineering, Donghua University, Shanghai, 201620 People’s Republic of China; 3 Sichuan Provincial Engineering Research Center of Functional Development and Application of High Performance Special Textile Materials, Chengdu Textile College, Chengdu, 611731 People’s Republic of China

**Keywords:** Perfect verticalization, Multiscale pores, Heat supply/insulation model, Cold/hot evaporation, Maximum utilization of energy

## Abstract

Hydrogel-based evaporators offer unique advantages for seawater desalination, yet the high verticalization of water transport channels remains significantly constrained by the random arrangement of polymers. Herein, a versatile and scalable “wet-spinning hydrogel fibers assisted with constrained alignment (HFCA)” strategy is proposed to fabricate hierarchically porous hydrogel fiber evaporator, achieving the perfectly vertical, large-scale spaces between adjacent fibers and small-scale pores within the hydrogel itself. The synergistic role of multiscale pores significantly enhances the siphon effect between fibers and the water transport capacity, while improving thermal localization, light absorption, and salt flux. Moreover, rather than employing the popular “heat isolation model”, a dual “heat supply/insulation model” is introduced for the first time in a three-dimensional evaporator, where a novel heating layer providing additional heat to the bulk water, along with an insulation layer minimizing heat loss. Together, these components create a positive net energy balance, transforming the single cold into a combined cold/hot evaporation manner on the side surface. Using this model, the HFCA evaporator exhibits the high evaporation rate (8.09 kg m^−2^ h^−1^, 1 KW m^−2^) among the reported hydrogel-based evaporators and achieves exceptional outdoor evaporation (64.74 kg m^−2^ for 9 h), accompanied with salt tolerance, anti-oil fouling, and self-cleaning capacities.

**Supplementary Information:**

The online version contains supplementary material available at 10.1007/s40820-026-02120-z.

## Highlights

- A versatile yet scalable “wet-spinning hydrogel fibers assisted with constrained alignment (HFCA)” strategy is proposed to achieve a highly vertical hydrogel fiber aggregate with multiscale pore structure.
- The dual “heat supply/insulation model” transforms the conventional single cold evaporation into cold/hot evaporation on the side surface for the first time, maximizing energy utilization of 3D evaporator.
- HFCA makes a breakthrough in verticalization of water transport channels and maximum utilization of energy, accompanied with superior salt tolerance, anti-oil fouling and self-cleaning ability.

**Supplementary Information:**

The online version contains supplementary material available at 10.1007/s40820-026-02120-z.

## Graphical abstract

**Figure:**
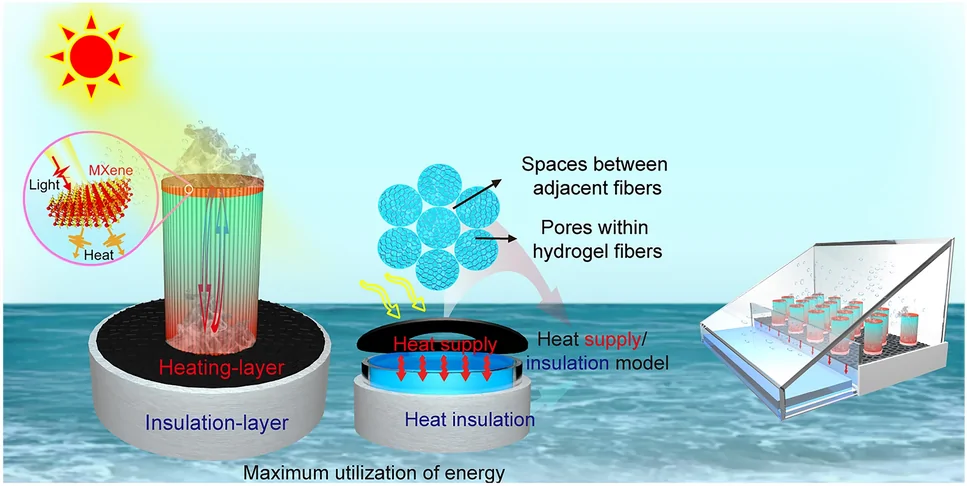

## Introduction

The scarcity of freshwater resources and the impact of climate change have made desalination technology an essential strategy to address the global freshwater crisis [1, 2]. While seawater desalination has advanced from early simple distillation to modern reverse osmosis technology, challenges such as environmental pollution, high energy consumption, and elevated costs continue to hinder its large-scale application. In recent years, the advent of solar-driven interfacial evaporation (SDIE) technology has emerged as a promising solution to these challenges [3, 4], SDIE employs photothermal conversion materials, including carbon-based and metal-based nano-ions, inorganic semiconductors, and other materials, to convert solar energy into thermal energy. This process involves photon absorption within the surface or internal structure of these materials [5, 6], facilitating the evaporation at the evaporator–water–air interface [7, 8]. Consequently, maximizing energy utilization efficiency [9], seawater evaporation efficiency, and salt-resistance stability has become a central focus in this field [10–12].

In SDIE systems, the evaporator substrate and its internal structure primarily determine the water transport capacity, making them critical components in influencing the overall efficiency of solar evaporation systems. Various evaporator substrates, such as aerogels [13], yarn [14], fabrics [15], and hydrogels [16], have been explored. Among these, hydrogels stand out owing to their three-dimensional polymer network, which is enriched with water molecules. This structure significantly reduces the evaporation enthalpy of water and enhances the efficiency of thermal energy utilization, making hydrogels one of the most ideal evaporator substrates for SDIE [17, 18]. However, the random arrangement of polymer chains and their disordered entanglements, inherent in hydrogels, often create irregularly curved channels. These irregular channels severely limit the water transport capacity in SDIE systems, resulting in the failure to efficiently return salt from the evaporated surface to the bulk water. This shortcoming leads to salt accumulation [19], which adversely affects the system’s efficiency and stability. To address these challenges, extensive research has focused on the verticalization of water channels. For example, directional freezing technology has demonstrated that aligning water transport pathways improves both the evaporation rate and salt resistance [20, 21]. Despite its effectiveness, directional freezing is limited to a narrow range of polymers (e.g., polyvinyl alcohol and alginate) and is constrained by the weak driving force of ice crystals, which makes it difficult to construct highly vertical channels. Thus, developing a versatile and robust approach to create perfectly vertical water transportation pathways with tunable dimensions is both urgent and challenging for hydrogel evaporators.

During the assembly of evaporative systems, many researchers adopt the popular “heat isolation model”, which incorporates a foam insulation layer between the evaporator and bulk water to minimize heat loss during transfer from top to bottom. This approach effectively localizes heat and reduces heat transfer to the bulk water [22–24], thereby improving the thermal efficiency of evaporators, particularly conventional two-dimensional (2D) designs. However, this single “heat isolation model” overlooks the advantages of the high surface area in emerging three-dimensional (3D) evaporators and the potential for steam escape from dark evaporation on the side surfaces. This limitation restricts the capture of additional energy from both the “3D evaporator from bulk water” and the “bulk water from the external environment” [25]. To maximize the benefits of 3D evaporators’ high surface area and fully activate the evaporation potential of their side surfaces, a versatile heat transfer/absorption model that addresses the contradiction between heat barrier and heat capture is urgently required [26]. In summary, creating a highly vertical pore structure, coupled with a rational heat transfer/absorption model, is expected to significantly enhance the evaporation performance of hydrogel-based 3D evaporators.

To tackle these challenges, this study proposes a “wet-spinning hydrogel fibers assisted with constrained alignment (HFCA)” strategy to achieve a highly vertical hydrogel fiber aggregate with a multiscale pore structure. Utilizing the rigid polymer chains of alginate hydrogels combined with MXene, strong and durable hydrogel fibers with a chemically and physically crosslinked double-network structure were developed. By applying a constraining force to a vertically arranged hydrogel fiber bundle, a vertically aligned hydrogel fiber evaporator with a multiscale pore architecture was fabricated. This design ingeniously created large-scale spaces between adjacent hydrogel fibers and small-scale pores within the hydrogel fibers. The synergistic effect of these highly vertical multiscale pores significantly enhanced the siphon effect between fibers, resulting in excellent water transport capability, a high evaporation rate, and rapid salt reflux. To overcome the contradiction between heat barrier and heat absorption in 3D evaporators, a versatile “heat supply/insulation model” was proposed. This model combines an “Oxford cloth with a honeycomb structure as a heating layer” and “porous foam as an insulation layer”. The heating layer efficiently is directly contact with the bulk water, absorbing solar energy and converting it into thermal energy to heat the bulk water. Simultaneously, the insulation layer is wrapped outside of the container full of the bulk water (rather than locating at the top surface of the bulk water) to avoid the heat loss of bulk water to surroundings. This innovative model interestingly transforms traditional cold evaporation into a combined cold/hot evaporation on the side surfaces of the 3D evaporator, enabling the evaporator to capture additional energy from both the high-temperature bulk water and the surrounding environment. Compared to the conventional “heat isolation model” (3 cm), the dual “heat supply/insulation model” dramatically increased the evaporation rate from 3.62 to 8.09 kg m^−2^ h^−1^. Outdoor evaporation reached 64.74 kg m^−2^ over 9 h (the maximum solar intensity was 0.67 KW m^−2^). The HFCA evaporator, with its multiscale vertical pores and innovative “heat supply/insulation model”, establishes a versatile and pioneering approach for designing 3D hydrogel evaporators with efficient energy utilization, high evaporation rates, and excellent salt resistance.

## Experimental Section

### Materials

Ti_3_C_2_T_*x*_ (MXene, 50 mg mL^−1^) was purchased from Jinan Sanchuan Co., Ltd., China. Sodium alginate (SA, 280 mPa s) was obtained from Qingdao Hai Zhi Lin Co., Ltd., China. Calcium chloride (CaCl_2_, 96%) was acquired from Sinopharm Chemical Reagent Co., Ltd., China. Glutaraldehyde (GA, 50%) was obtained from Shanghai Macklin Biochemical Co., Ltd., China. All the chemical reagents used met the analytical purity standard and could be applied directly in experiments without additional purification treatment.

### Synthesis of Hydrogel Fibers

First, 4.2 g of SA powder was incrementally mixed into 84 mL deionized water in three separate additions. The mixture was then vigorously stirred at 350 rpm using an electric mixer until the SA powder was completely dissolved, and then the initial defoaming was carried out at 200 rpm for 1 h. Subsequently, 6 mL of MXene suspension was introduced into the sodium alginate solution, and the stirring process was resumed for an additional 30 min to ensure thorough mixing. Upon completion of the stirring, the solution underwent ultrasonication for 60 min to facilitate secondary defoaming. The resultant SA/MXene spinning solution was then transferred into a syringe. Utilizing a micro-syringe pump set at a speed of 0.6 mm s^−1^, the spinning stock solution was extruded through the spinneret needle, forming a fine stream that was directed into a 10 wt% CaCl_2_ coagulation bath to induce the formation of a homogeneous hydrogel. Throughout the fiber generation and collection process, no tensile force was applied. Following this step, the fibers were subjected to secondary cross-linking with glutaraldehyde for a period of 8 h, after which they were soaked and washed in deionized water to complete the fabrication process.

### Assembly of Dual Heat Supply/Insulation Model

Alg_Ca/GA/MXene hydrogel fibers with a diameter of 0.8 mm were cut into uniform lengths of 4 cm (the height of the HFCA evaporator was 3 cm) and placed into the polyethylene ring (diameter of 1.35 cm). There was a slight deformation of the fibers near the rings, which was negligible due to the small number of roots. In the same way, Alg_Ca/GA hydrogel fibers without MXene were prepared and assembled in an evaporator.

### Characterizations

The morphology of both the surface and cross section for the freeze-dried hydrogel fibers were collected by scanning electron microscopy (SEM, EVO18, ZEISS, Germany), and the elements of the surface were characterized using energy-dispersive X-ray spectroscopy (EDS). Under the ATR mode, the Fourier transform infrared spectra (FTIR, Nicolet iS10, USA) were collected with the scanning times of 32 and a resolution of 2 cm^−1^. Elemental analysis of the hydrogel fibers was characterized by X-ray photoelectron spectroscopy (XPS, Escalab 250 Xi, Thermo Scientific, USA). Raman microscopy using laser wavelength of 532 nm was used to determine the physical interaction of the materials and the water state within the range of 3000–4000 nm. The hydrophilic contact angle and underwater oleophobic properties of the hydrogel fiber evaporator were tested using an optical contact angle meter (OCA 15EC). Optical transmission and reflectance of the hydrogel fibers at 280–2500 nm were measured using UV–visible near-infrared spectroscopy (UV-3600) with an integrated sphere. According to Kirchhoff’s law, the absorbance (A) of a hydrogel can be calculated indirectly from the reflectance (R) and transmittance (T) by the formula A = 1-R-T. This method evaluates the efficient light absorption of hydrogel fibers. The concentrations of metal ions in seawater and purified water before and after evaporation were measured by inductively coupled plasma-optical emission spectrometry (ICP-OES, Avio 200, Perkin Elmer).

### Solar Steam Test

Three distinct models were positioned on a high-precision electronic balance (accurate to 0.1 mg), which was interfaced with a computer. The SPDC software was employed to record the weight changes of water for the evaporator over a period of 1 h. A xenon lamp equipped with an AM1.5G filter, serving as a solar simulator (CEL-PE300L-3A), was utilized to simulate solar radiation under light conditions. After establishing the required simulated solar light intensity, the power output of the solar simulator and the distance between the light source of xenon lamp and the evaporator were adjusted. The density of optical power received on the surface of evaporator was determined by repositioning the auxiliary detector (CEL-NP2000). Throughout the experiments, the ambient temperature was maintained between 29 and 30 °C, with relative humidity of approximately 60%–75%. An infrared thermal camera was used to measure the temperature changes of surface for the evaporator. Typically, for a three-dimensional evaporator, the evaporated area (*Ae*) is equivalent to the light projection area (*Ap*), and the water evaporation rate of the evaporator is calculated using the following equation:

1 $$\begin{array}{*{20}c} {V = \frac{1}{{{\mathrm{Ae}}}}\frac{{{\mathrm{d}}m}}{{{\mathrm{d}}t}} = \frac{M}{{{\mathrm{Ap}}}}} \\ \end{array}$$

where *m* represents the mass of evaporated water at certain duration time of light (t). *M* is the slope of curve of water loss obtained by linear regression analysis, representing the mass of evaporated water per unit of time under steady-state condition. *Ae* (or *Ap*) is the surface area of the evaporator (area of light).

### Numerical Simulation

COMSOL calculation results for pressure and the equivalent heat transfer model of porous media for HFCA-10 evaporator [42].

Water transport process in the hydrogel fiber evaporator with highly vertical channels follows the Darcy flow:

2 $$\begin{array}{*{20}c} {\frac{{\partial \varepsilon _{p} \rho }}{{\partial t}} + \nabla \cdot \left( {\rho _{f} u} \right) = Q_{m} } \\ \end{array}$$

3 $$\begin{array}{*{20}c} {u = - \frac{k}{\mu }\left( {\nabla p - \rho g} \right)} \\ \end{array}$$

where $\epsilon_{{\mathrm{p}}}$ is the porosity, ρ is the density of the liquid (kg m^−3^), t is the time (s), u is the Darcy velocity, and $Q_{m}$ is the evaporation mass flow rate. k is the permeability of the evaporator,μ is the viscosity of the water (Pa s), and p is the pressure (Pa). Pressure is mainly determined by capillary forces: $P = 2\sigma \cos \left( \theta \right)/R$, where σ is the surface tension of the liquid, *θ* is the contact angle, and *R* is the average equivalent radius (It can be analogized to the half distance between the fibers).

The equivalent heat transfer model of porous media was used to describe the heat transfer process in HFCA evaporator:

4 $$\begin{array}{*{20}c} {\left( {\rho C_{p} } \right) _{eff} \frac{\partial T}{{\partial t}} + \rho_{f} C_{p,f} u \cdot \nabla T + \nabla \cdot q = Q} \\ \end{array}$$

5 $$\begin{array}{*{20}c} {q = - k_{{{\mathrm{eff}}}} \nabla T} \\ \end{array}$$

6 $$\begin{array}{*{20}c} {\left( {\rho C_{p} } \right)~_{{{\mathrm{eff}}}} = \varepsilon _{p} \rho _{f} C_{{p,f}} + \theta _{s} \rho _{s} C_{{p,s}} + \left( {1 - \theta _{s} } \right)\rho _{f} C_{{p,f}} } \\ \end{array}$$

7 $$\begin{array}{*{20}c} {k_{{{\mathrm{eff}}}} = \varepsilon _{p} k_{f} + \theta _{s} k_{s} + \left( {1 - \theta _{s} } \right)k_{f} + k_{{{\mathrm{disp}}}} } \\ \end{array}$$

where $\left( {\rho C_{p} } \right) _{{{\mathrm{eff}}}}$ is the effective volumetric heat capacity at constant pressure, ${\mathrm{Q}}$ is the heat source, $k_{{{\mathrm{disp}}}}$ is the thermal dispersion coefficient, and $k_{{e{\mathrm{ff}}}}$ is the effective thermal conductivity. The $\theta_{s}$ is the volumetric fraction of solids, the fluid volume fraction is the porosity of the evaporator $\theta_{f} = \left( {1 - \theta_{s} } \right)$.

## Results and Discussion

### Design Principle of the HFCA Evaporator and Heat Supply/Insulation Model

**Fig. 1:**
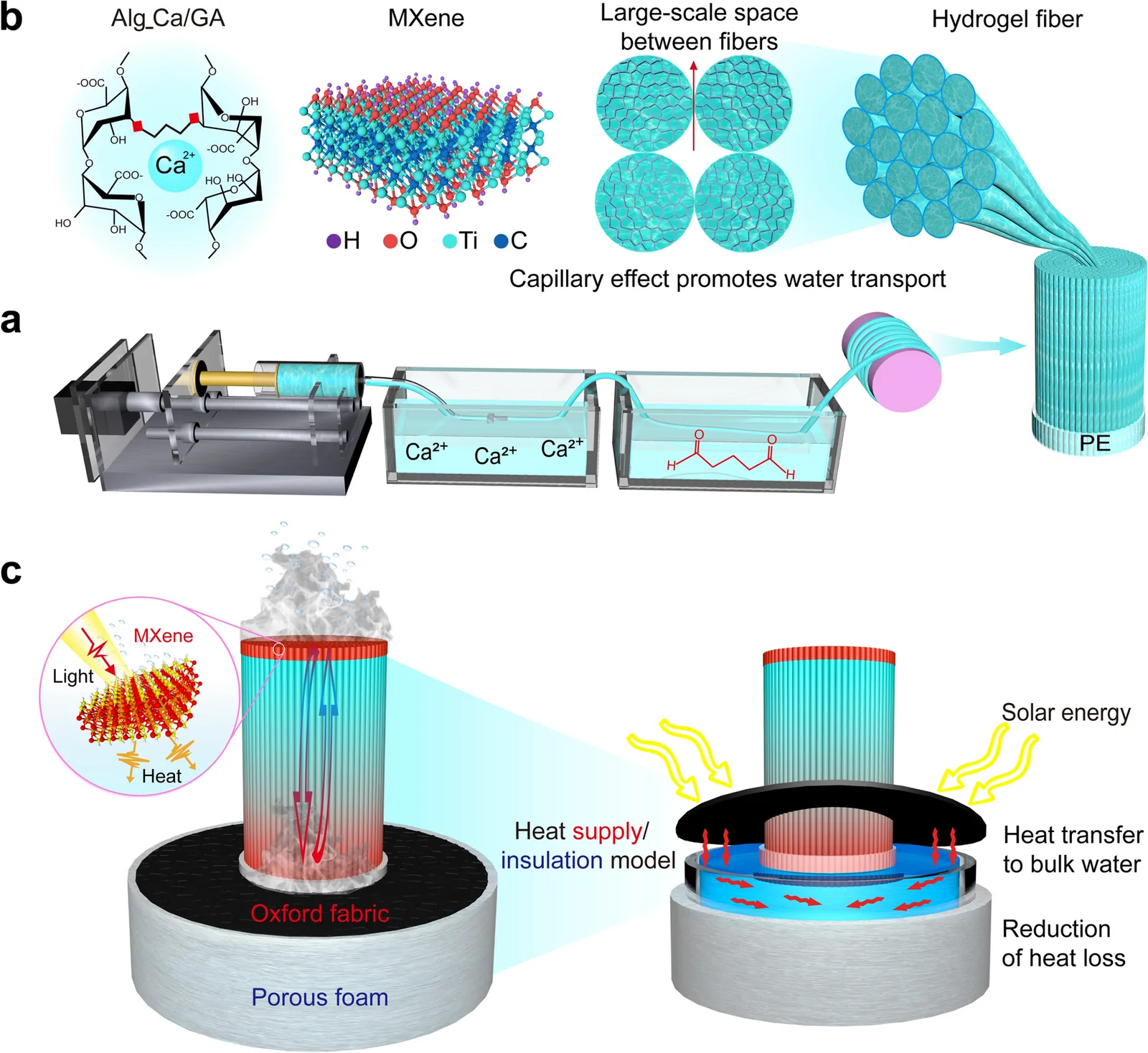
3D hydrogel fiber evaporator with highly vertical and multiscale pore structure. **a** Wet-spinning process to prepare strong hydrogel fibers. **b** Multiscale pore structure and the chemical structure of hydrogel fiber. **c** Assembly of dual “heat supply/insulation model”

The “wet-spinning hydrogel fibers assisted with constrained alignment” strategy and the versatile “heat supply/insulation model” proposed in this study are illustrated in Fig. 1. A natural polymer-based hydrogel fiber was continuously fabricated using a simple yet well-established wet-spinning technology. The MXene exhibits a theoretical photothermal conversion efficiency approaching 100%, indicating a perfect energy conversion [5]. Alginate, characterized by its rigid polymer chains, was selected and further in situ incorporated with MXene. The hydrogel fibers were strengthened and toughened through a combination of physical coagulation (using Ca^2+^) and chemical coagulation (using glutaraldehyde), resulting in calcium alginate/MXene hydrogel fibers (Alg_Ca/GA) with enhanced mechanical properties (Fig. 1a, b). A vertically arranged hydrogel fiber evaporator was subsequently fabricated by applying a constraining force to align the hydrogel fibers. The rigid polymer chains (G segments) and the physical–chemical dual cross-linking network of the alginate hydrogel fibers ensured a highly vertical alignment of the fibers under the applied force. This process successfully constructed perfectly vertical channels with multiscale dimensions for the 3D hydrogel fiber evaporator. Notably, the use of highly aligned hydrogel fibers facilitated the creation of highly vertical and uniform spaces between adjacent fibers, overcoming the limitations of traditional evaporators, which typically suffer from irregular and uneven channels. In addition, the evaporator featured small-scale pores within the hydrogel fibers themselves. The precise regulation of hydrogel fiber characteristics was achieved by adjusting parameters such as the precursor solution concentration, the diameter of the spinneret hole, and the injection speed (Fig. S1a–c). The large-scale space between neighboring fibers was tunable over a wide range by varying the magnitude of the constraining force. In this study, based on the forming kinetics and rheological properties of the hydrogel fiber, the optimal parameters were determined to be a precursor solution concentration of 5 wt%, a spinneret hole diameter of 1 mm (the diameter of the hydrogel fibers was 0.8 mm), and a syringe pump propulsion speed of 0.6 mm s^−1^. To further ensure the structural integrity and vertical alignment of the hydrogel fibers, a polyethylene (PE) ring was used to constrain the fibers into a stable and oriented arrangement. This approach ensured the overall structural stability of the evaporator.

An unprecedented dual “heat supply/insulation model” was designed to optimize the evaporation process on the side surface and maximize energy utilization in the 3D evaporator system (Fig. 1c). In this model, the “Oxford cloth with a honeycomb structure” was directly contact with the bulk water, absorbing solar energy and converting it into thermal energy to heat the bulk water. In addition, a “porous foam insulation layer” was wrapped outside of the container full of the bulk water to avoid the heat loss of bulk water to surroundings. This combination enabled the hydrogel fiber aggregate evaporator to operate in a combined cold/hot evaporation mode on the side surface. The highly vertical, multiscale pore structure played a crucial role in this system by enabling efficient bottom-up water transport while optimizing thermofluid dynamics. This configuration facilitated the vertical transfer of heat absorbed from the high-temperature bulk water through the evaporator, accelerating thermal evaporation from the bottom via the side channels. During evaporation, heat absorption and heat loss caused the evaporator channel temperature to drop below the ambient temperature. Consequently, the evaporator harnessed additional energy from the surroundings to drive cold evaporation. On the top surface, the unique electromagnetic wave absorption properties of MXene were utilized to absorb a broad spectrum of visible and near-infrared sunlight. The nanostructure of MXene further induced the localized surface plasmon resonance effect, where heat generated from absorbed solar energy was dissipated to the surroundings through lattice vibration and scattering. This significantly enhanced the efficiency of light energy capture and conversion into thermal energy [27]. These combined effects of wide-spectrum light absorption and efficient photothermal conversion enabled rapid evaporation at the top surface–water–air interface in the 3D evaporator.

It is noteworthy that the proposed “wet-spinning HFCA” strategy employed a highly industrialized wet-spinning technique, which is extendable using a spinneret for the tailored and large-scale fabrication of hydrogel fibers (Fig. S1d–f). The HFCA fabrication process is simple, easily controllable, and scalable.

### Multiscale Structure of HFCA Evaporator

**Fig. 2:**
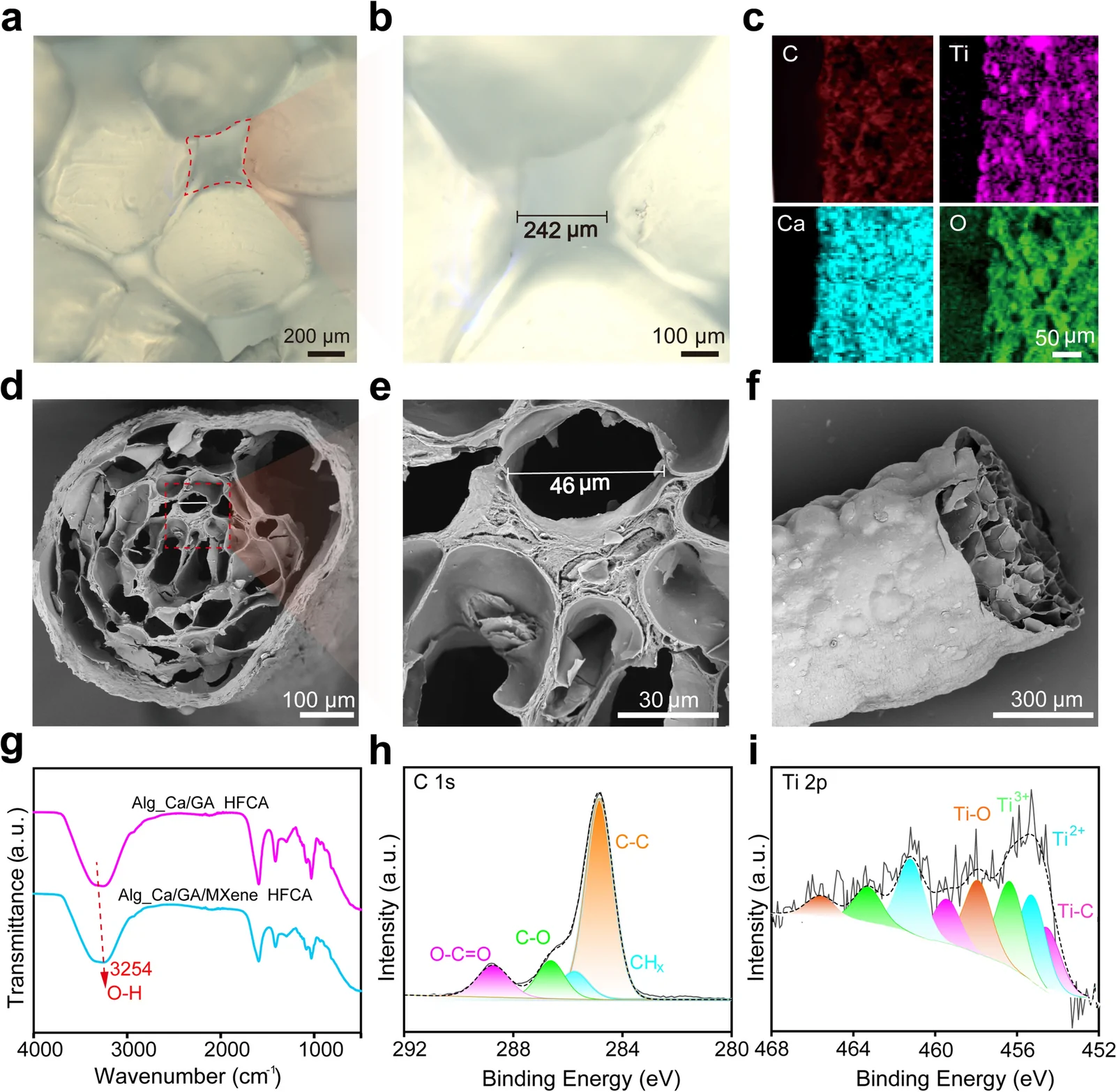
Multiscale structure of HFCA evaporator.** a**,** b** Optical microscope images of large-scale spaces between adjacent fibers of HFCA-10 evaporator.** c**–**f** SEM images of EDS elemental analysis of surfaces and the pore structure within the hydrogel fibers. **g** FTIR spectra of Alg_Ca/GA and Alg_Ca/GA/MXene hydrogel fibers.** h**,** i** High-resolution XPS spectra of C 1*s* and Ti 2*p* in HFCA

The multiscale structure of the HFCA evaporator was systematically revealed in Fig. 2. First, in order to clearly observe the large-scale spaces between adjacent hydrogel fibers, the cross section and surface morphology of the HFCA evaporator without MXene were collected using an optical microscope, eliminating the barrier effect of MXene on incident light. According to the real morphology, the hydrogel fibers were regular and tightly aligned under the external constraining force (Figs. 2a and S2a), forming large-scale spaces between neighboring fibers with relatively uniform size of 200–300 μm (Fig. 2b). More importantly, the directional alignment of stiff fibers made the large-scale spaces high verticalization available (Fig. S2a). Notably, the size of the large-scale spaces can be widely regulated by adjusting the magnitude of the external constraining force (Fig. S2b, c). Secondly, the hydrogel itself exhibits a porous structure with rich water molecules (Fig. 2d–f), which contains multiscale pores with size of 20–110 μm (Figs. 2d, e and S2d) and very small pores with size of 1–15 μm located at pore-wall structure (Figs. 2d and S2e). The high swelling ratio (SR = 819%) and saturated water content (SWC = 89.1%) of the hydrogel fibers further demonstrated their high water content character and porosity (Figs. 2e, S2f and Note S1). In summary, our proposed HFCA evaporator characterized with both highly vertical large-scale spaces between adjacent fibers and small-scale pores within hydrogel fiber. The highly vertical multiscale pore structure significantly enhanced the siphon effect and capillary action, which could facilitate efficient transport capacity of water, heat, and salt within the evaporator and thus promote rapid evaporation.

Additionally, the selected calcium alginate/MXene hydrogel matrix exhibited a flat and smooth surface (Fig. 2f) under freeze-dried state, indicating the uniform distribution of MXene and tight connection between mutual phases. According to elemental analysis of the surface (Fig. 2c), the uniformly distributed Ca element proved that the wet-spinning process obtained a homogeneous Ca^2+^-based cross-linked network, the distribution of Ti element proved the uniform distribution of MXene in hydrogel matrix. According to the Fourier transform infrared spectroscopy (FTIR), the absorption peak located at 3258 cm^−1^ was attributed to the stretching vibration of hydroxyl group (–OH), which shifted to lower wavelength upon compounding with MXene, indicating that hydrogen-bonding interaction occurred between MXene and calcium alginate (Fig. 2g). This is attributed to the abundance of carboxylic acid (–COOH) and –OH groups in alginate chains, which can form hydrogen bonds with the terminal –O and –OH groups of MXene. Concurrently, X-ray photoelectron spectroscopy (XPS) technology was employed to analyze the bonding and surface structural changes of neat MXene and Alg_Ca/GA/MXene hydrogel fiber (Fig. S3a), and the appearance of Ti element in the full XPS spectrum of hydrogel fiber confirmed the successful introduction of MXene [28]. Compared with MXene, the disappearance of the C–Ti peak in the HFCA C 1*s* spectra (Figs. 2h and S3b) demonstrated that the alginate chains physically interacted with the MXene nanosheets. For the Ti 2*p* spectra (Figs. 2i and S3c), the peaks of Ti^2+^ and Ti^3+^ decreased slightly from 455.5 and 456.6 eV (MXene) to 455.2 and 456.3 eV (hydrogel fiber), which may be due to the formation of ionic bonds between Ca^2+^ and MXene nanosheets. Furthermore, new peaks of Ca 2*p*_3/2_ (347.7 eV) and Ca 2*p*_1/2_ (351.28 eV), as well as Cl 2*p*_3/2_ (198.3 eV) and Cl 2*p*_1/2_ (199.9 eV) were observed in the HFCA spectrum (Fig. S3d, e), which further proved that calcium chloride as a coagulation bath promoted the preparation of hydrogel fibers and its chelating effect with polymer chains. Consequently, the strong hydrogen-bonding interaction between MXene and alginate not only ensured the uniform distribution of MXene in the hydrogel fiber matrix and the tight connection between two phases, but also played a secondary cross-linking role in alginate chains. The dual roles mentioned above ensured the uniform dispersion of the spinning raw solution, the continuity of wet-spinning (Video S1), and the stability of the hydrogel fibers.

In summary, this research successfully constructed highly vertical large-scale spaces between hydrogel fibers and small-scale pores within hydrogel fiber for 3D hydrogel evaporator. The multiscale pore structure, coupled with the abundant polar groups on the surface of the alginate chains/MXene, would play a significant role in the management of water transport for the evaporator. Meanwhile, the high strength and toughness of the hydrogel fiber along with constraining force would ensure the mechanical stability of the evaporator during long-term application. For instance, the hydrogel fiber with a diameter of 0.8 mm could endure a load of 150 g without breaking (Fig. S4a). The tensile strength could reach to 4 MPa, and the fracture toughness was 2.34 MJ m^–3^ (Fig. S4b).

### Water Transport and Thermal Management in HFCA Evaporator

**Fig. 3:**
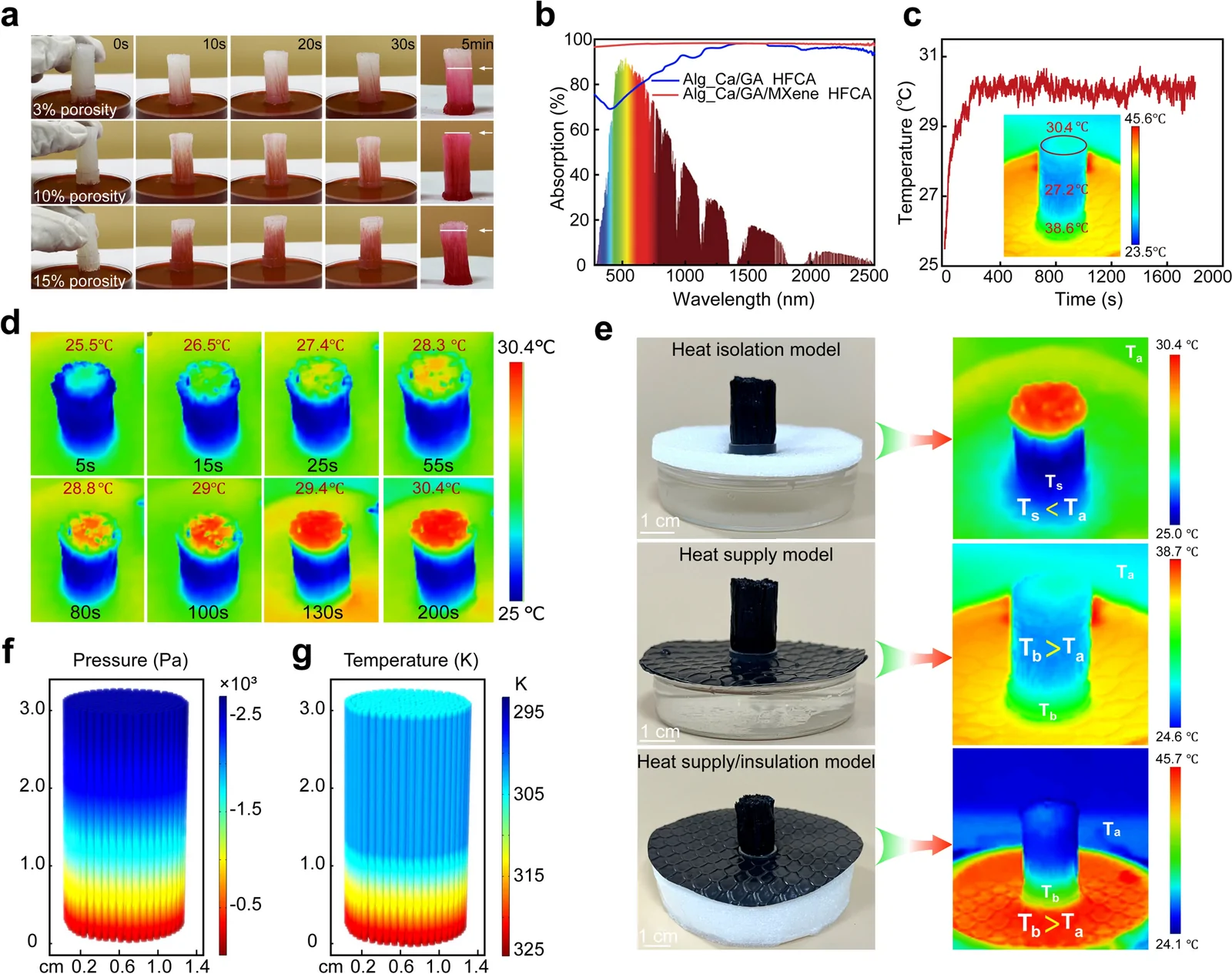
Water transport and thermal management of HFCA evaporator. **a** Height of water transport for HFCA evaporator with porosity of 3%, 10%, and 15%. **b** UV–vis–NIR absorption spectra of the Alg_Ca/GA and Alg_Ca/GA/MXene evaporators.** c**,** d** Variation of temperatures at the top surface of the evaporator with water in the large-scale spaces under one simulated solar intensity. **e** Numerical and infrared thermal images of the “heat isolation model”, “heat supply model”, and “heat supply/insulation model”. ** f**,** g** COMSOL calculation results for pressure and the equivalent heat transfer model of porous media for HFCA-10 evaporator

The efficient water transport capacity of the 3D SDIE hydrogel fiber evaporator ensures the continuous supply of water during the interfacial evaporation, which is a critical factor in optimizing the evaporation kinetics of the evaporator. First, the hydrophilicity of the hydrogel fibers attracts water molecules through physical interactions, such as hydrogen bonding, which promotes the adsorption and diffusion of water on surface of the fiber. Water contact angle analysis (Fig. S5) revealed that water droplets were completely absorbed within only 103 ms after contacting HFCA, indicating the superior hydrophilic properties of the hydrogel fibers in this study. Second, for the highly vertical multiscale pore architecture proposed in this research, small-scale pores provide hydrogel fibers with high content of water to maintain the low evaporation enthalpy, and reasonable large-scale spaces significantly enhances the siphon effect between fibers and the water transport capacity. Here, the siphon effect originates from the strong adhesive force exerted by hydrogel fiber walls on bulk water, forming continuous liquid film bridges between adjacent fibers. Under the influence of surface tension, this liquid film generates a negative pressure gradient, driving bulk water to continuously ascend through macroscopic pores between fibers. This is the dominant factor in determining the water transport process. Accordingly, our research achieved precise size regulation of the large-scale space structure, that is porosity of the evaporator, by adjusting the external constraining force of PE rings to the hydrogel fiber aggregate, producing evaporators with porosity of 3%, 10%, and 15% (Note S2), respectively (named as HFCA-3, HFCA-10, and HFCA-15). The transport height of dyed water in HFCA evaporators without MXene (Figs. 3a and S6) showed that the water transport height first increased and then decreased with decreasing porosity at the same height (3 cm) (Video S2), in which the HFCA-10 evaporator exhibited the best water transport capability. When the porosity of the evaporator was adjusted from 15% to 10%, the driving negative pressure increased due to the decreased large-scale space, leading to a higher water transport height within the evaporator. It should be noted that the siphon effect between hydrogel fibers was influenced by various factors, including surface nature and the structure of the fiber aggregate. Therefore, when the porosity of the evaporator was further reduced to 3%, the excessive external binding force and the corresponding too small space increased viscous resistance, hindering the upward transport of dyed water, significantly diminishing both the height and efficiency of water transport. Hence, the comprehensive performance of the evaporator with an optimal porosity of 10% was systematically investigated in this research.

Regarding the capability of light absorption of the evaporator (Fig. 3b), compared with HFCA evaporator without MXene, the Alg_Ca/GA/MXene HFCA evaporator exhibited broad and efficient light absorption (~ 98%) across the wavelength range of 280–2500 nm, accompanied with extremely low reflectance (~ 2%) and transmittance (~ 0%), demonstrating that the introduction of MXene significantly enhanced the light absorption. Meanwhile, the HFCA evaporator with large-scale pore channels in this study further extended the propagation path of incident light (Fig. S7a), which promoted multiple scattering and absorption of sunlight, effectively enhancing the capture ability of incident light. Furthermore, the photothermal conversion performance of the interface for evaporator is pivotal in improving the evaporation rate. According to the temperature change under one simulated solar irradiation intensity (1 KW m^−2^), the HFCA evaporator without water in the large-scale spaces responded rapidly, and the interface temperature increased rapidly to 31.9 °C for 250 s (Fig. S7b). Even in actual operation of evaporator full of water, the interface temperature could reach about 30.4 °C (Fig. 3c, d). This phenomenon confirmed the outstanding capability of photothermal conversion for HFCA evaporator. This phenomenon confirmed the outstanding capability of photothermal conversion for HFCA evaporator. This is attributed to the excellent conductivity and the abundant terminal groups (–OH, –O) of MXene, exhibiting superior capability of electromagnetic wave absorption. When the frequency of incident light matches with plasma resonance frequency of MXene, localized surface plasmon resonance will be generated. In this way, MXene could efficiently capture solar energy and then convert it into thermal energy, enhancing photothermal conversion efficiency. The solar-to-vapor conversion efficiency (η) at the evaporative interface of the HFCA evaporator can be calculated by the following equation [45]:

8 $$\begin{array}{*{20}c} {\eta = \frac{{\dot{m}H_{{\mathrm{e}}} }}{{C_{{{\mathrm{opt}}}} q_{0} }}} \\ \end{array}$$

9 $$\begin{array}{*{20}c} {\dot{m} = m - m_{d} } \\ \end{array}$$

where *η* is the photothermal conversion efficiency, $\dot{m}$ is the evaporation rate due to light irradiation, *m* is the total evaporation rate, *m*_*d*_ is the dark evaporation rate, *H*_e_ is the evaporation enthalpy of water, *C*_opt_ is the optical concentration of the solar simulator, and *q*_*0*_ is the intensity of the solar irradiation (1 KW m^−2^). The solar-to-vapor conversion efficiency of the HFCA at one solar irradiation intensity was calculated to be 90%.

Optimizing thermal conductivity is the key aspect to improve thermal management of the evaporator. According to the analysis of thermal conductivity, the hydrogel fiber evaporator without water between fibers had a lower thermal conductivity of 0.1927 W m^−1^ K^−1^ (Fig. S8) due to low thermal conductivity (approximately 0.026 W m^−1^ K^−1^) of air within large-scale space between the fibers. Moreover, to simulate the solar evaporation condition, the HFCA evaporator full of water between fibers maintained a low thermal conductivity (0.5641 W m^−1^ K^−1^). Notably, the vertical channel made the heat dissipation in the lateral direction of evaporator reduced (Fig. 3d). The preferential heat conduction behavior in the HFCA evaporator originates from the structural anisotropy formed by the vertically arranged hydrogel fiber channels. The vertically aligned hydrogel fibers form continuous solid–liquid channels along the thickness direction, facilitating the axial conduction of heat to the evaporation interface. In contrast, lateral heat dissipation is significantly hindered by the discontinuous solid contacts, the thermal resistance increases notably. Furthermore, according to the thermodynamic principle of minimum entropy production, heat spontaneously transfers along paths with “high demand and low resistance.” The vertical channels precisely provide such an optimal path, enabling heat to preferentially accumulate at the evaporation interface rather than diffusing to the lateral environment with no evaporation demand. The thermal localization capability of HFCA is predominantly attributed to its vertically aligned fiber configuration, discontinuous thermal pathways, and multiscale porous air-containing network. This design not only enhanced the local thermal effect on the evaporator surface, but also confirmed the advantage of HFCA evaporators in thermal localization. Such thermal localization was crucial for enhancing the performance of solar evaporators, as it significantly reduced the dissipation of heat through precise heat management. This ensured that majority of the absorbed solar energy was directly utilized to heat the interfacial liquid, demonstrating excellent capability of thermal management.

For the assembly of 3D evaporation systems, the single “heat isolation model” is a popular approach, whereby the evaporator is separated from the bulk water by a thermal insulation layer, leading to the side surface temperature of evaporator (*T*_s_) being lower than the ambient temperature (*T*_a_) (Fig. 3e). Although this model reduces heat loss via heat conduction, convection, and radiation during evaporation, it seriously diminishes the advantage of capturing additional energy for “3D evaporator from bulk water” and “bulk water from the external environment.” In fact, except for the photothermal conversion in the interface of evaporator, making full use of the wide spectrum of solar radiation should be also considered to increase the potential evaporation on the side surface of the 3D evaporator. Accordingly, this research proposed an innovative “heat supply/insulation” combined model, which incorporated the Oxford cloth with honeycomb structure as heating layer between the 3D evaporator and bulk water. It could efficiently absorb solar energy and convert it into thermal energy, thereby providing heat to the bulk water. Then, the high-temperature bulk water was covered with a porous foam as insulation layer, avoiding its heat loss to the surroundings. The dual heat supply/insulation model combines the heat absorption of the Oxford cloth layer, which captures solar energy not utilized by traditional evaporation methods, with the insulation layer that prevents heat loss to the surroundings. This enables the evaporator to efficiently utilize both solar energy and thermal energy from the bulk water, significantly improving the overall evaporation rate and energy efficiency. In contrast to the traditional “heat isolation model”, both the “heat supply model” and “heat supply/insulation model” resulted in a higher bottom side surface temperatures (*T*_b_) than the ambient temperature (*T*_a_) (Fig. 3e). This was because, in accordance with the second law of thermodynamics, the energy tended to transfer from high-temperature regions to low-temperature regions. With the Oxford cloth heating the bulk water, the heat spontaneously transferred from the bulk water to the evaporator, transforming the traditional cold evaporation into hot evaporation on the bottom side surface. With the proceed of the evaporation induced heat absorption and heat loss, the evaporator temperature decreased below the ambient temperature, leading to the absorption of additional energy from the surroundings for evaporator. To further validate the aforementioned mechanism, the inter-fiber distance was calculated based on the macroscale spatial configuration, and a corresponding model was established (Fig. S9). COMSOL Multiphysics finite element simulation was employed to investigate the pressure distribution and temperature field of the evaporator, with simulations of flow velocity and heat transfer conducted based on Darcy’s law and porous media fluid heat transfer theory. The simulation results revealed that the HFCA-10 evaporator generates negative surface pressure (Fig. 3f), which together with the siphon effect between the fibers drive water transport within the vertical channels. Additionally, the temperature distribution of the HFCA-10 evaporator was numerically simulated using the equivalent heat transfer model of porous media (Fig. 3g), where the simulation results exhibit qualitative consistency with the experimental results in terms of trend and spatial distribution. The aforementioned results demonstrate that the reasonable design of vertical channels and the distance between adjacent fibers is crucial for promoting water transport and photothermal conversion of the evaporator.

### High-Efficient Evaporation of Dual Heat Supply/Insulation Model

**Fig. 4:**
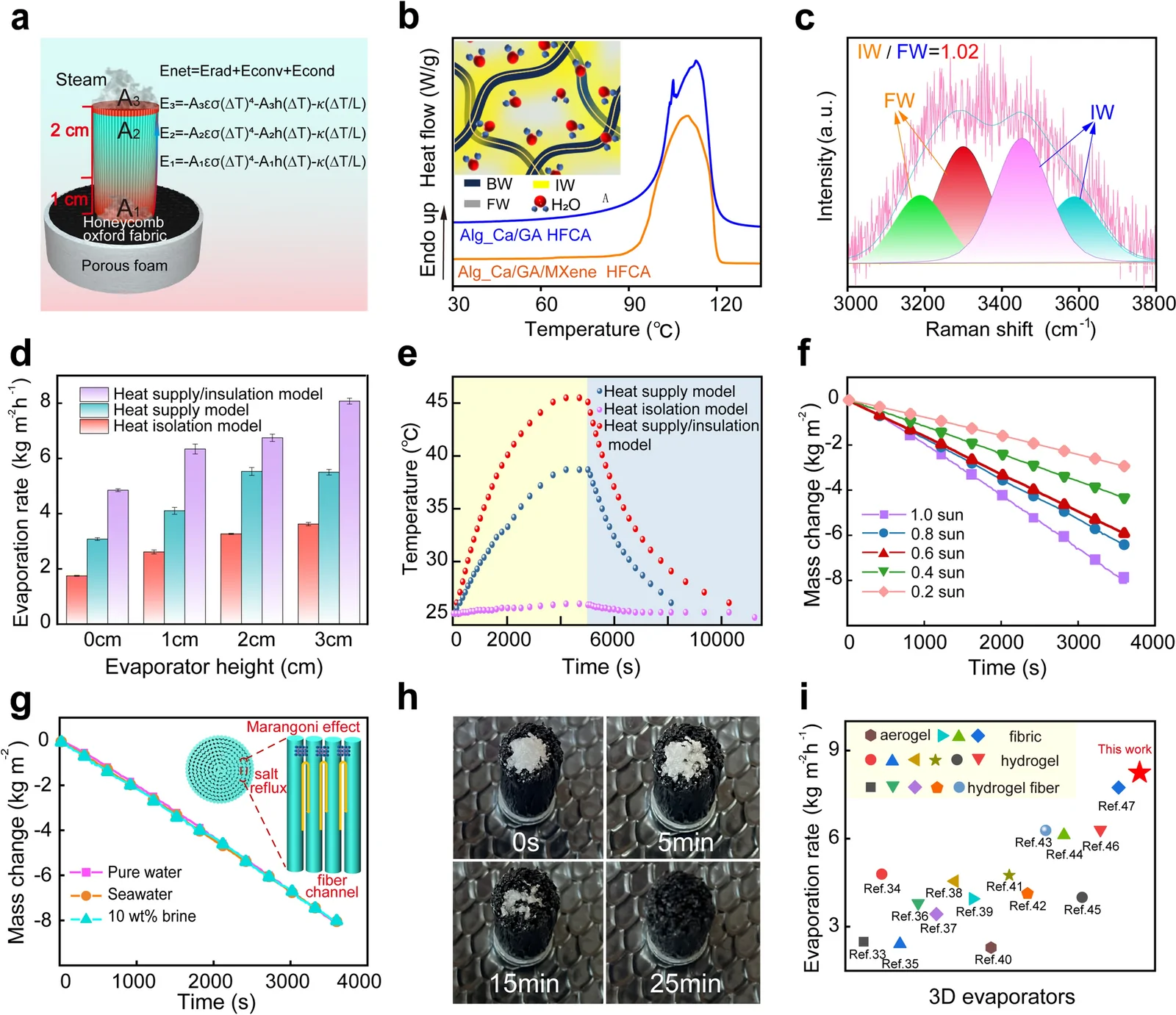
Evaporation performance of “heat supply/insulation model”-based evaporators. **a** Schematic setup of the solar evaporation test. **b** DSC curves of Alg_Ca/GA and Alg_Ca/GA/MXene hydrogel fibers. **c** Raman spectra of water in the Alg_Ca/GA/MXene HFCA evaporator. **d** Variation of evaporation rates in different evaporation model. **e** Temperature variation of bulk water in different evaporation models. **f** Evaporation rate of Alg_Ca/GA/MXene HFCA-10 evaporator under different solar illumination. **g** Evaporation rate of Alg_Ca/GA/MXene HFCA-10 evaporator in salt solution. **h** Self-cleaning dissolution of NaCl on the surface of the Alg_Ca/GA/MXene HFCA-10 evaporator. **i** Comparison of evaporation rates for Alg_Ca/GA/MXene HFCA-10 evaporator with other 3D evaporators

Reducing the evaporation enthalpy of water molecules and enhancing the efficiency of thermal energy utilization are unique advantages of hydrogel-based evaporators in the realm of desalination. The results in this research also revealed (Fig. 4b) that the evaporation enthalpy of Alg_Ca/GA hydrogel fiber is significantly lower than that of pure water (2270 J g^−1^), confirming that the hydrogel can indeed diminish the evaporation enthalpy of water. The corresponding mechanism originates from three states of water molecule induced by the mutual interaction between water molecules and polymer network, including free water (FW), intermediate water (IW), and bound water (BW) [17]. Here, the BW is those physically interacted strongly with hydrophilic groups of the polymer network. While FW are far away from hydrophilic groups of the polymer network, retaining structural character akin to bulk water. In contrast, intermediate water (IW) possesses weaker and more dynamic hydrogen bonds due to partial coordination with polymer functional groups. This weaker hydrogen-bond network decreases the average hydrogen-bond energy per water molecule, thereby lowering the overall evaporation enthalpy compared to bulk water dominated by strongly hydrogen-bonded clusters [29]. Particularly, the incorporation of MXene into the hydrogel further reduced the evaporation enthalpy of Alg_Ca/GA/MXene evaporator (Fig. 4b). This phenomenon was possibly due to that the incorporation of MXene introduces abundant terminal groups (–O and –OH) that form competitive hydrogen bonds with the groups of alginate chains with the polar groups of alginate chains (–COOH, –OH) (Fig. 2g). This competition weakens the polymer–water hydrogen-bonding network and disrupts the continuity of hydrogen-bond clusters, promoting the formation of more IW species with higher molecular mobility and lower binding energy. According to the Raman spectroscopy analysis of water states for Alg_Ca/GA/MXene evaporator (Fig. 4c), the absorption peaks at 3189 and 3300 cm^−1^ correspond to FW, while the peaks at 3452 and 3587 cm^−1^ are assigned to weakly hydrogen-bonding IW. The ratio of IW/FW = 1.02 indicated that more water molecules were weakly bound to polymer chains, which significantly reduced latent heat during water evaporation [30]. Therefore, the synergistic effect of the polymer–MXene interaction and the redistribution of water molecular states jointly contribute to weakening the overall hydrogen-bonding network, lowering the latent heat of evaporation, and enhancing the thermal energy utilization efficiency of the hydrogel-based evaporator.

To investigate the influence of the dual “heat supply/insulation model” proposed in this research on the overall energy of the evaporator, the distribution of temperature for 3D evaporator was in situ monitored (Fig. 4a). The data revealed that under one-sun illumination, the average temperature of region *A*_1_ and *A*_3_ within the “heat supply/insulation model”-based evaporator surpassed ambient temperatures, of which the temperature difference would result in the energy loss from these regions to the surroundings through heat radiation and convection. In contrast, region *A*_2_ exhibited a temperature lower than that of the ambient, which led to the capacity of absorbing energy from the environment. This is because the heat supply layer of the “heat supply/insulation model”-based evaporator provided heat to the bulk water, while the insulation layer prevented heat dissipation of the bulk water as compared to the conventional “heat isolation model” based evaporator. Consequently, the evaporator can theoretically also gain energy from the bulk water through heat conduction. Throughout the evaporation process, the net energy of the evaporator could be calculated using the following equation [31, 32]:

10 $$\begin{aligned} E_{e} & = - A_{1} \varepsilon \sigma \left( {T_{1}^{4} - T_{e}^{4} } \right) - A_{2} \varepsilon \sigma \left( {T_{2}^{4} - T_{e}^{4} } \right) - A_{3} \varepsilon \sigma \left( {T_{3}^{4} - T_{e}^{4} } \right) \\ & \quad - A_{1} h\left( {T_{1} - T_{e} } \right) - A_{2} h\left( {T_{2} - T_{e} } \right) - A_{3} h\left( {T_{3} - T_{e} } \right) - E_{{{\mathrm{cond}}}} \\ \end{aligned}$$

where *A*_1_, *A*_2_, and *A*_3_ are areas of hot evaporation on the side surface (~ 4.27 cm^2^), cold evaporation on the side surface (~ 8.54 cm^2^), and the top interface (~ 1.45 cm^2^), respectively. *T*_1_ (38.6 °C), *T*_2_ (27.6 °C), and *T*_3_ (30.4 °C) are the corresponding average temperature of three regions. *T*_e_ (29.6 °C) is the average temperature of surrounding environment. *ε* is the surface emissivity (~ 0.9) of evaporator. *σ* is the Stefan–Boltzmann constant, with a value of 5.67 × 10^−8^ W m^−2^ K^−4^. *h* is the convective heat transfer coefficient, with a value of 10 W m^−2^ K^−1^. *E*_cond_ is the heat conduction energy (details of the calculation are from Note S3, Supporting Information). Calculation results showed that the radiation and convection energy loss from the *A*_1_ region to surroundings was 0.02275 and 0.03849 W, respectively. Region *A*_2_ absorbed energy from the surroundings, with radiation and convection gains of 0.00957 and 0.0170816 W, respectively. The radiation and convection energy loss from the *A*_3_ region to the surroundings were 0.0006605 and 0.00116 W, respectively. Additionally, the evaporator gained conduction energy from the bulk water, amounting to 0.0395 W. Therefore, the net energy gained from the surroundings during the operation of the solar evaporator was 0.0085 W. These calculations demonstrated that although the hot evaporation on side surface in the “heat supply/insulation model” incurred radiation and convection energy loss, its unique advantage of heat supply led to an increase in total conduction energy. This positive net energy balance confirms that the model can utilize environmental energy to compensate for losses, thereby maintaining a positive overall energy balance for the evaporator. This further validates the superior thermophysical performance of the dual “heat supply/insulation model.”

The evaporation rate serves as a pivotal metric for assessing the performance of an evaporator, dictating its evaporation efficiency in the desalination process. Under one-sun illumination, the evaporation rates of HFCA-3, HFCA-10, and HFCA-15 evaporators were 7.71, 8.09, and 7.0 kg m^−2^ h^−1^, respectively (Fig. S11a), further demonstrating the excellent water transport capacity with 10% porosity enhanced the evaporation rate. Furthermore, the evaporation rate of three kinds of evaporation model escalated with increasing height of evaporator (Fig. 4d). This is because for 3D evaporators with the same projected areas, the side surface provides an additional water/air interface, which became pounced with increasing height of evaporator, thereby substantially boosting the evaporation rate (Fig. S10). Concurrently, compared with the single “heat isolation model” (3.62 kg m^−2^ h^−1^) and the single “heat supply model” (5.49 kg m^−2^ h^−1^)-based evaporators, the dual “heat supply/insulation model”-based evaporator achieved an evaporation rate of 8.09 kg m^−2^ h^−1^ at a height of 3 cm (Fig. 4d), showing a significant advantage of this combined model (Table S1, The heat supply and heat insulation strategies are optional, not required for core operation). The remarkably high evaporation rate of the “heat supply/insulation model” based evaporator indicated that the heating layer was conducive to the heat absorption and the hot evaporation of the evaporator by heating the bulk water, further underscoring the superiority of the foam as insulation layer in reducing heat loss of the bulk water. Moreover, the evaporation rate of the Alg_Ca/GA/MXene HFCA evaporator significantly surpassed that of the Alg_Ca/GA HFCA evaporator without MXene (Fig. S11b), further proving the positive role of MXene with wide-range absorption of light and efficient photothermal conversion in facilitating evaporation in the interface of evaporator. Furthermore, the superiority of the dual “heat supply/insulation model” is also reflected in extending the duration of high-evaporation-rate state. Under one-sun illumination, the stable temperature of bulk water for the “heat isolation model”, “heat supply model”, and “heat supply/insulation model” were recorded as 26.0, 38.7, and 45.6 °C, respectively (Fig. 4e). Upon cessation of simulated solar irradiation, the heat supply model sustained a high evaporation rate for 3 h (Fig. S11c) before gradually stabilizing, enhancing the potential of the evaporator for night-time applications. Additionally, most current researches focused on using excessively high solar irradiance intensity (2, 3, 4, and 5 KW m^−2^) to highlight the excellent evaporation performance of evaporators. However, lower sun illumination was a regular phenomenon in practical applications. For instance, the maximum solar irradiance in most regions rarely exceeded 0.8 KW m^−2^, making the high evaporation rate of evaporative systems at low solar light intensity particularly important. Accordingly, a systematic study of the evaporation rates of the dual “heat supply/insulation model”-based evaporator at low solar insolation intensity (0.8, 0.6, 0.4, and 0.2 KW m^−2^) was carried out (Fig. 4f). The data revealed that with the decreasing intensity of solar light, the evaporation rate corresponded to 8.09 (45.6 °C), 6.45 (39.8 °C), 5.92 (35.3 °C), 4.36 (33 °C), and 2.95 (30.2 °C) kg m^−2^ h^−1^ (Fig. S11d). Notably, the evaporation rate of the “heat supply/insulation model” at a simulated solar irradiance of 0.4 KW m^−2^ (4.36 kg m^−2^ h^−1^) already surpasses that of the “heat isolation model” at 1 KW m^−2^ (3.62 kg m^−2^ h^−1^). In addition, the evaporation rate of the HFCA evaporator remains essentially constant in different environments (Fig. S12). These results further demonstrate the superiority of the dual “heat supply/insulation model” over the conventional single “heat isolation model”, showing great potential in natural environment applications.

The salt-resistance stability of the evaporator is crucial in practical applications, as salt crystallization can hinder sunlight from reaching the surface of evaporator, weakening the capacity of capturing/converting solar energy for evaporator and thus decreasing the overall photothermal conversion efficiency. Additionally, salt crystallization may block water transport channels and steam escape pathways, affecting the working duration of the desalination. According to the evaporation rates of the HFCA-10 evaporator in brines with different concentrations, under one-sun illumination, the evaporation rates of evaporator in pure water, 3.5 wt% brine, and 10 wt% brine were 8.09, 8.04, and 8.02 kg m^−2^ h^−1^, respectively (Fig. 4g). During short-term tests under one-sun illumination, the evaporation rate of the HFCA-10 evaporator showed only a slight variation among pure water, 3.5 wt% brine, and 10 wt% brine (Fig. 3a), suggesting that the vertically aligned porous channels facilitated continuous water transport and alleviated the short-term effect of increased salt concentration on evaporation (Fig. S13a). However, during long-term operation, the gradual accumulation of salt at the interface led to a decline in evaporation rate. After ten cycles (equivalent to 10 h) of continuous evaporation, the evaporation rates for the 3.5% and 10% brine concentrations during the tenth cycle were 7.98 and 7.27 kg m⁻^2^ h⁻^1^, respectively (Fig. S13b, c), corresponding to average evaporation rates over the ten cycles of 7.8 and 7.4 kg m⁻^2^ h⁻^1^. The reduced evaporation rate was due to that the rapid water evaporation during long time leaved some salt particles at the evaporator interface. The absence of salt crystals on the surface (Fig. S14) was attributed to the concentration gradient of salt was formed between the interface of evaporator and the bulk water at the bottom. The Marangoni effect drove rapid water flow in the vertical channels, while water reflux transported salts from the evaporation area back into the bulk water, keeping the salt concentration on the evaporation surface below saturation and thus preventing the crystallization of salt. This mechanism is enabled by the highly vertical and continuous channels, which facilitate rapid convective salt reflux. In addition, based on the evaporation rate under simulated alternative day and night conditions of practical operation, using 10 wt% brine as an example, the evaporation rate of the HFCA-10 evaporator decreased from 8.02 to 7.27 kg m^−2^ h^−1^ after 10 h, which could return to 7.95 kg m^−2^ h^−1^ the next day after 10 h of dark evaporation (Fig. S13c). During night evaporation with low evaporation rate, the convection and diffusion of water within the large-scale pores endowed the salt particles that stayed at the evaporation interface flow back into the bulk water (Video S3). This proved the strong self-cleaning ability and durability of evaporator. In addition, the evaporator was operated continuously for 15 days (6 h per day, 1 KW m^−2^) in seawater with a very small drop in evaporation rate (Fig. S13d), demonstrating its stability for prolonged evaporation. As evidenced by XPS analysis, after 15 days of operation, the HFCA evaporator showed an increased peak area ratio of Ti–O and TiO_2_, while the characteristic Ti–C peak remained prominent (Fig. S15a–c). This confirms that MXene in the evaporator underwent only partial oxidation, without severe degradation of its core structure. Furthermore, scanning electron microscopy images revealed no salt accumulation on the hydrogel fiber walls or within the fibers (Fig. S15d–f). Collectively, these results suggest that the slight decrease in evaporation rate may be attributed to the partial oxidation of MXene and the accumulation of trace salts between fibers.

This further proved that the highly vertical water transport channels endowed the evaporator with strong self-cleaning capability, stability, and durability. To further evaluate the ability of salt dissolution and reflux for HFCA evaporator, the salt dissolution behaviors of evaporators with different porosities were investigated in the presence NaCl particles on the top surface. The results showed that with the same weight of NaCl particles, the salt particles on the surface of HFCA-10 evaporator were significantly reduced within 5 min, further completely dissolving into the bulk water for 25 min. However, the salt particles on HFCA-3 and HFCA-15 only exhibited the phenomenon of surface wetting (Figs. 4h and S16). Thus, Alg_Ca/GA/MXene HFCA-10 evaporator presented excellent salt solubility and salt blocking properties. This phenomenon further confirmed that the reasonable distance between neighboring fibers induced the strongest siphon effect, which provided the largest speed of water transport with highly vertical channels, definitely facilitating the rapid dissolution of salt particles. Consequently, compared to the reported 3D evaporators (Fig. 4i and Table S2) [33–47], the vertically multiscale porous HFCA evaporator in this research, characterized with the low evaporation enthalpy of hydrogel matrix, the highly vertical water transport channels, and the efficient energy utilization of the dual “heat supply/insulation model” exhibits a distinct advantage of evaporation rate.

### Practical Application of HFCA Evaporator

**Fig. 5:**
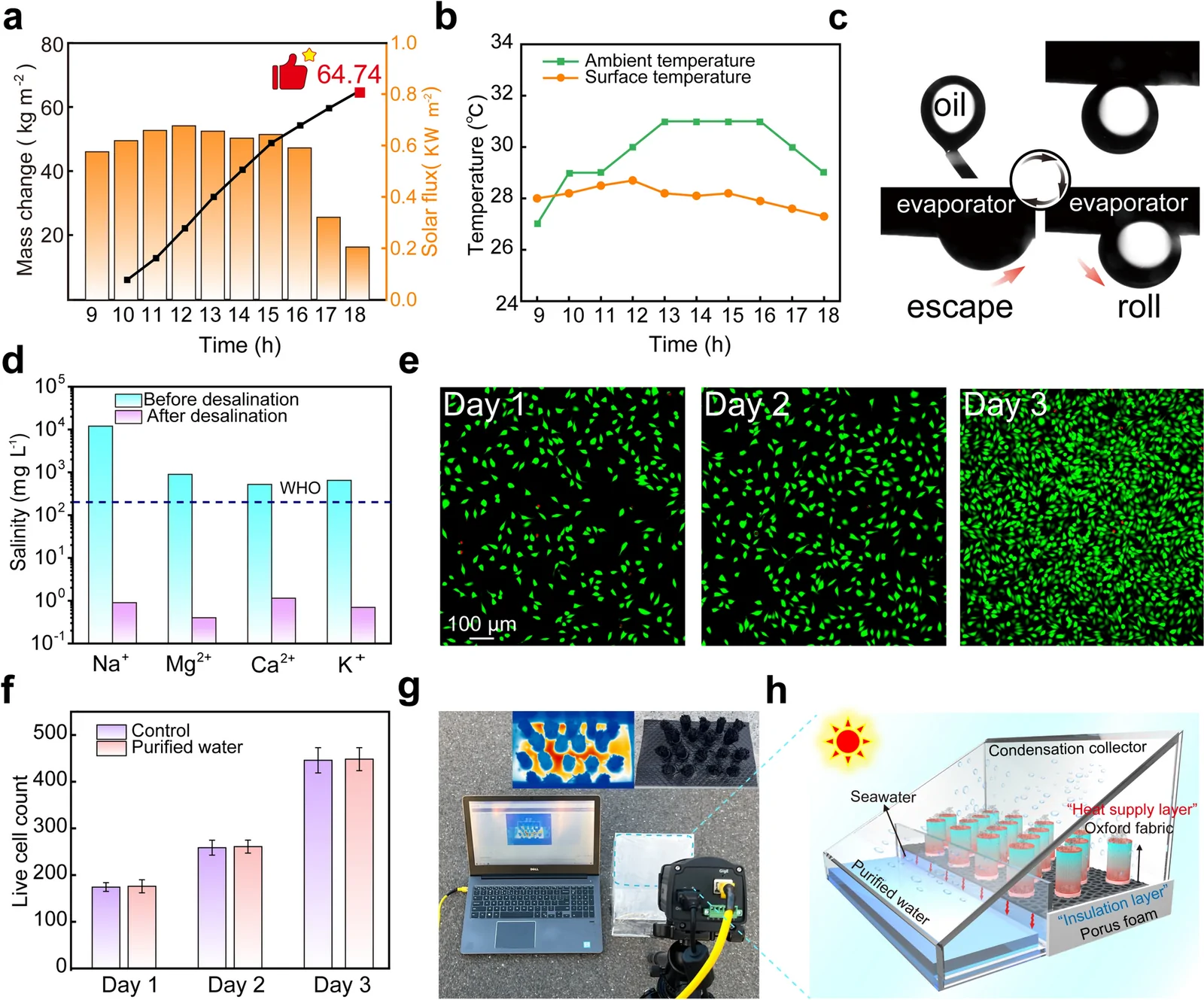
Practical application of HFCA evaporator and its great potential in large scale. **a** Solar flux and mass change in outdoor environment. **b** Ambient temperature and interface temperature of light-heat conversion for HFCA-10 evaporator in outdoor. **c** Anti-oil performance of HFCA-10 evaporator in underwater. **d** Concentration of main cations before and after evaporation.** e**,** f** Images of fibroblasts cultured and the number of fibroblasts proliferating in the purified water from HFCA-10 evaporator.** g**,** h** Large-scale array assembly of HFCA evaporators

The HFCA evaporator with the dual “heat supply/insulation model” proposed in this research demonstrated outstanding evaporation performance in practically environment. The HFCA evaporator combined with the “heat supply/insulation model” evaporation system was placed in an inclined condensate collection device (Fig. S17). Data collected from the outdoor evaporation of the HFCA evaporator in seawater (Qingdao, Yellow Sea, China) showed that the cumulative evaporation reached 64.74 kg m^−2^ with the time range of 9:00–18:00, a maximum sun intensity of 0.67 KW m^−2^ and an average ambient temperature of 29.8 °C (Fig. 5a, b). Notably, the average evaporation rate of the evaporator (7.19 kg m^−2^ h^−1^) in outdoor exceeded the value of in-laboratory (5.92 kg m^−2^ h^−1^, 0.6 KW m^−2^), which was attributed to the inclined irradiation of outdoor sunlight, which increased the light-receiving area of the evaporator compared to the vertical irradiation in the laboratory. Additionally, the Oxford cloth with honeycomb structure as heating layer efficiently absorbed solar energy and provided heat for bulk water, while the porous foam as insulation layer avoided heat loss of bulk water to the surroundings. The bulk water accumulated heat over time, enhancing the hot evaporation on the side surface of the evaporator. These combined effects mentioned above increased the evaporation rate of the whole evaporation system.

A large amount of oil and organic substances are existed in seawater due to the industrial emissions and leaks of cruise ship. Therefore, the capability of oil resistance for evaporator is crucial for ensuring its efficient operation in long term and the purity of purified water. Here, oily organic matter (1,2-dichloroethane) was employed to simulate oil contamination in seawater, in which the contact angle between oil and surface of HFCA-10 evaporator was 141°. Moreover, the oil rapidly rolled and then escaped after contacting the surface of HFCA evaporator (Fig. 5c), indicating that the evaporator possessed excellent oleophobic property. This is attributed to the numerous polar groups (–COOH, –OH) in the alginate chains and the terminal –O, –OH groups of MXene, which could form strong hydrogen bonds with water molecules, creating a stable physical barrier of water layer and thus exhibiting super-hydrophilic/oleophobic properties. Further, the vegetable oil became oleophobic contact angle with the evaporator surface, and the convective diffusion of water in the large-scale fiber voids rapidly occurred rolling escape (Video S4). The small-scale pores inside the hydrogel fibers with high water content and the siphon effect of the vertical channels between the fibers enhance the convection and diffusion of water (Video S3), which together prevented the oil contamination from entering the evaporator space.

Moreover, the dyes from dyeing and finishing factories are usually observed in natural seawater. It was found that the anionic dye of methyl orange (MO) (Fig. S18a) and the cationic dye of methyl blue (MB) (Fig. S18b) became clean and transparent after evaporation through the HFCA evaporator. The UV–vis spectroscopy further confirmed that the colored contaminants were not detected in this purified water (Fig. S18), demonstrating the capability of efficient dye removal for the HFCA evaporator. Therefore, the HFCA evaporator can remain stable operation in various harsh environments.

For purified water collecting from natural seawater after evaporation by the HFCA-10 evaporator, compared to natural seawater, it showed a significant reduction in the concentrations of major cations (Na^+^, Mg^2+^, Ca^2+^, and K^+^) with three orders of magnitude, reaching the standard of drinking water for the World Health Organization (WHO) (Figs. 5d and S19a). Therefore, using the method of fixing the distance between electrodes, the resistivity of purified water was 4.83 MΩ. This value was between the resistivity of deionized water (7.29 MΩ) and domestic water (2.24 MΩ), and was much higher than that of seawater (293.4 KΩ) (Fig. S19b). These results confirmed the outstanding capability of removing ions for HFCA evaporator upon desalination process. For instance, the purified water with very low ion concentration could be directly used for crop seed cultivation and irrigation. The seeds soaking by this purified water germinated and grew over time, whereas no growth was observed for the same seeds kept in seawater (Fig. S20). Fibroblast cells were cultured using the purified water collected from the HFCA-10 evaporator and double-distilled water as solvents (Figs. 5e, S21 and Note S4). For both purified water and double-distilled water, live/dead cell staining observed a large number of live cells with green fluorescence, and the fibroblast cells proliferated over time with similar average cell counts (Fig. 5f), further demonstrating the purity and uniformity of the purified water [48]. Therefore, the purified water obtained from the HFCA evaporator possessed excellent purity, which could meet different kinds of requirements in both production and daily life.

A “propulsion–solidification–coiling collection” device was arranged using a microfluidic spinning machine (Fig. S1e), a coagulation bath vessel, and a microfluidic reactor, achieving integrated process of preparation and collection of hydrogel fibers (Video S1). To further exploit the advantages of the combined cold/hot evaporation mechanism on the side surfaces, an array-based configuration was adopted instead of increasing the diameter of individual evaporators (Fig. 5g). Specifically, a series of HFCA evaporator units were further arranged in an array within a sloped condensation collection device (Fig. 5g, h). This configuration could realize the absorption of light with very wide areas, fully exerting the advantages of combined cold/hot evaporation model of the side surface. This modular configuration enables the expansion of evaporation output through structural assembly, thereby offering a practical and controllable approach for extending the evaporation area without compromising the underlying cold/hot evaporation synergy. It should be noted that the design of heat supply/insulation models has certain limitations in practical application scope, making them difficult to deploy directly in natural water bodies for long-term operation. Consequently, they are primarily suited to small-scale, controllable water treatment scenarios.

## Conclusion

Based on the proposed “wet-spinning hydrogel fibers assisted with constrained alignment” strategy, the HFCA evaporator with highly vertical multiscale pores was successfully constructed, in which the large-scale spaces between neighboring fibers could be adjusted in a wide range by changing the magnitude of constraining force around the hydrogel fiber aggregates. With the optimal porosity of 10%, the highly vertical pores between adjacent fibers significantly enhanced the siphon effect, improving water transport capability. Meanwhile, these vertical channels further promoted multiple scattering and absorption of light, which enhanced photothermal conversion efficiency and ensured that the heat of the evaporator was only localized on its surface irradiated by the simulated sunlight, thus facilitating the excellent capability of thermal management. Furthermore, a combined “heat supply/insulation model” was innovatively introduced for the first time, achieving an unprecedented evaporation mode of combined cold/hot evaporation on the side surface of 3D evaporator. Here, the Oxford cloth with honeycomb structure as heating layer could efficiently absorb solar energy and provide heat for the bulk water, while the porous foam as insulation layer could minimize heat loss. Using this model, the bottom of the 3D evaporator absorbed heat from the high-temperature bulk water, transforming the traditional cold evaporation into a combined cold/hot evaporation on the side surface. Therefore, the net energy gained from the surroundings during the operation of the solar evaporator was 0.0085 W. With the design of the highly vertical hydrogel fiber aggregate with multiscale pore structure and the dual “heat supply/insulation model”, the evaporator with a height of 3 cm achieves an evaporation rate of 8.09 kg m^−2^ h^−1^ under one-sun illumination, with a cumulative evaporation of 64.74 kg m^−2^ after 9 h of continuous outdoor evaporation. Under the highly vertical large-scale spaces, the siphon effect between fibers was remarkably enhanced, the evaporator showed excellent self-cleaning ability without salt crystallization on the surface for 10 h of continuous evaporation in 10 wt% brine. Moreover, the evaporator exhibited outstanding anti-oil fouling performance. This work represents a significant breakthrough in the verticalization of transport channels and the maximization of energy utilization for hydrogel-based evaporators. We believe these versatile concepts will drive transformative developments in hydrogel materials, wearable sensors, and water desalination technologies, capturing the immediate interest of a broad range of researchers in these fields.

## Supplementary Information

Below is the link to the electronic supplementary material.

Supplementary file1 (MP4 12609 KB)

Supplementary file2 (MP4 6520 KB)

Supplementary file3 (MP4 5.21 MB)

Supplementary file4 (MP4 2078 KB)

Supplementary file5 (DOCX 10.5 MB)
