# Supplementary material for: Hydrogel Fiber Evaporator with Vertical Channels Integrated with Dual Heat Supply/Insulation Model for Continuous Solar Desalination
Source: Nanomicro Lett. 2026 Feb 28;18:261. doi: 10.1007/s40820-026-02120-z (PMC12950158; doi:10.1007/s40820-026-02120-z)
Supplement: Supplementary file 5 — Supplementary file5 (DOCX 10.5 MB) [file 40820_2026_2120_MOESM5_ESM.docx]

Supporting Information for

**Hydrogel Fiber Evaporator with Vertical Channels Integrated with Dual Heat Supply/Insulation Model for Continuous Solar Desalination**

Tian Wang^1^, Shuai Gao^1^, Yongli Yu^1^, Zhigang Chen^2,^*, Lili Wang^1,^*, Xiansheng Zhang^1,3,^ *

^1^ Shandong Key Laboratory of Medical and Health Textile Materials, College of Textiles and Clothing, State Key Laboratory of Bio-Fibers and Eco-Textiles, Qingdao University, Qingdao 266071, P. R. China

^2^ State Key Laboratory of Advanced Fiber Materials, College of Materials Science and Engineering, Donghua University, Shanghai 201620, P. R. China

^3^ Sichuan Provincial Engineering Research Center of Functional Development and Application of High Performance Special Textile Materials, Chengdu Textile College Chengdu 611731, P. R. China

*Correspondence authors. E-mail: [zgchen@dhu.edu.cn](mailto:zgchen@dhu.edu.cn) (Zhigang Chen); [llwang@qdu.edu.cn](mailto:llwang@qdu.edu.cn) (Lili Wang); [xshzhang@qdu.edu.cn](mailto:xshzhang@qdu.edu.cn) (Xiansheng Zhang)

**Note S1 Calculation of Expansion Ratio and Saturated Water Content**

The water content of the hydrogel fibers was evaluated by comparing their dry weight and the equilibrium weight after swelling. The swelling ratio (*SR*) and saturated water content (*SWC*) were calculated using the following equations [S1]:

$$\begin{aligned} \text{SR=}\frac{\text{m}_{\text{s}}\text{-}\text{m}_{\text{d}}}{\text{m}_{\text{d}}}\text{×100\%} \end{aligned}\text{(}\text{S}\text{1)}$$

$$\begin{aligned} \text{SWC=}\frac{\text{m}_{\text{s}}\text{-}\text{m}_{\text{d}}}{\text{m}_{\text{s}}}\text{×100\%} \end{aligned}\text{(}\text{S}\text{2)}$$

where *m_s_* is the equilibrium weight of the hydrogel fiber after the swelling and *m_d_* is the weight of the hydrogel fiber after complete drying. The average values from multiple measurements were used, and the *SR* and *SWC* (Figure S2e, Supporting Information) of the hydrogel fiber were calculated to be 819% and 89.1%, respectively. These results indicated that the hydrogel fiber involved rich water and corresponding internal porous structure.

**Note S2 Calculation of Evaporator Porosity**

The porosity of the HFCA evaporator is calculated by the ratio of the total area of the fibers to the total area of the rings:

$$\begin{aligned} P=\frac{\pi R^{2}-n\pi r^{2}}{\pi R^{2}}\times100\%\#\text{(}\text{S}\text{3)} \end{aligned}$$

where *P* is the porosity of the HFCA evaporator, *R* is the radius of the circle, *r* is the radius of the hydrogel fibers, and *n* is the number of hydrogel fibers within the PE ring.

**Note S3 Heat Transfer from High-Temperature Bulk Water to Evaporator**

The infrared images of the “heat supply/insulation model” elucidated substantial difference in the longitudinal temperature gradient throughout the evaporation process, which was predominantly attributed to the cold/hot evaporation on the side surface of the HFCA evaporator. To tackle the nonlinear heat conduction predicament between the high-temperature bulk water and the evaporator, a segmented calculation was implemented. This approach entailed dissecting the entire heat conduction process into more manageable segments, presupposing approximate linear heat conduction within each segment. Consequently, the overall heat conduction process could be divided into three distinct segments: the initial segment corresponding to the hot evaporation region, the subsequent corresponding segment to the cold evaporation region, and the terminal segment corresponding to the interface evaporation region. The density of heat flow in every region was calculated using the following equation [S2]:

$$\begin{aligned} \text{J}_{\text{cond}}\text{=κ}\left( \frac{\text{∆T}}{\text{L}} \right)\text{\#} \end{aligned}\text{(}\text{S}\text{4)}$$

where *κ* is the thermal conductivity of the HFCA-10 evaporator (0.1927 W m^-1^ K^-1^), *∆T/L* is the temperature gradient along the longitudinal direction in the different zones. The temperature gradients in the zones *A*_1_, *A*_2_, and *A*_3_ are 1310, 250, and 160 K m^-1^ respectively. Thus *J*_1cond_, *J*_2cond,_ and *J*_3cond_ are about 252.437, 48.175, and 30.832 W m^-2^ respectively.

$$\begin{aligned} \text{E}_{\text{cond}}\text{=}\text{J}_{\text{cond}}\text{∙A} \end{aligned}\text{(}\text{S}\text{5)}$$

where *A* is the cross-sectional area of the conduction path (1.45×10^-4^ m^2^). The *E*_1cond_, *E*_2cond_, and *E*_3cond_ are approximately 0.03665, 0.0074 and -0.00447 W, respectively. *E*_1cond_ and *E*_2cond_ are the energy gained from the high-temperature bulk water and *E*_3cond_ is the energy lost from the top interface to the cold evaporation on the side surface of HFCA.

**Note S4 Fibroblast Culture**

**Materials.** Purified water (Experimental collection), DMEM high-glucose medium powder (Procell, China), Fetal Bovine Serum (Procell, China), Penicillin and streptomycin (Procell, China), sodium bicarbonate (Macklin, China), Calcein/PI cell viability and cytotoxicity detection kit (Beyotime, China), Cell Counting Kit-8 (MCE, USA), L929 cells (Procell, China).

**Preparation of Culture Medium.** 0.67 g of DMEM high sugar medium powder was accurately weighed and dissolved in 50 mL of purified water. After the powder was completely dissolved, 0.185 g of sodium bicarbonate (analytically pure) powder was added to the solution and stirred until completely dissolved. Then the pH of the solution was adjusted to 7.2. Subsequently, the solution was filtered under positive pressure using filter membranes with a pore size of 0.2 μm to remove any bacteria.

**Cell Culture.** L929 cells were cultured in a DMEM high glucose medium containing 10% fetal bovine serum and 1% of penicillin and streptomycin, which were maintained in a controlled incubator at 37°C with 5% CO₂ (All DMEM medium was prepared from purified water unless otherwise specified).

**Cytocompatibility Assay.** To assess the biosafety of purified water, L929 cells were seeded in 96-well cell culture plates at a density of 5×10^3^ cells per well and cultured in DMEM medium. Cytotoxicity was analyzed using the Cell Counting Kit-8 (CCK-8) and the live/dead cell staining kit. 100 μL of DMEM medium and 10 μL of CCK-8 solution were added to each well on every day, respectively. Following incubation at 37 °C for 1 h, the absorbance value of the solution was measured at 450 nm using an enzyme marker. Similarly, live/dead cell staining reagents were added to the wells every day according to the instructions of manufacturer, and the wells were incubated for 30 min at 37 °C in the dark. The images were collected using a fluorescence microscope (Nikon, Japan). ImageJ software was used to calculate the cell density per unit area at each point in time.

**Supplementary Figures**


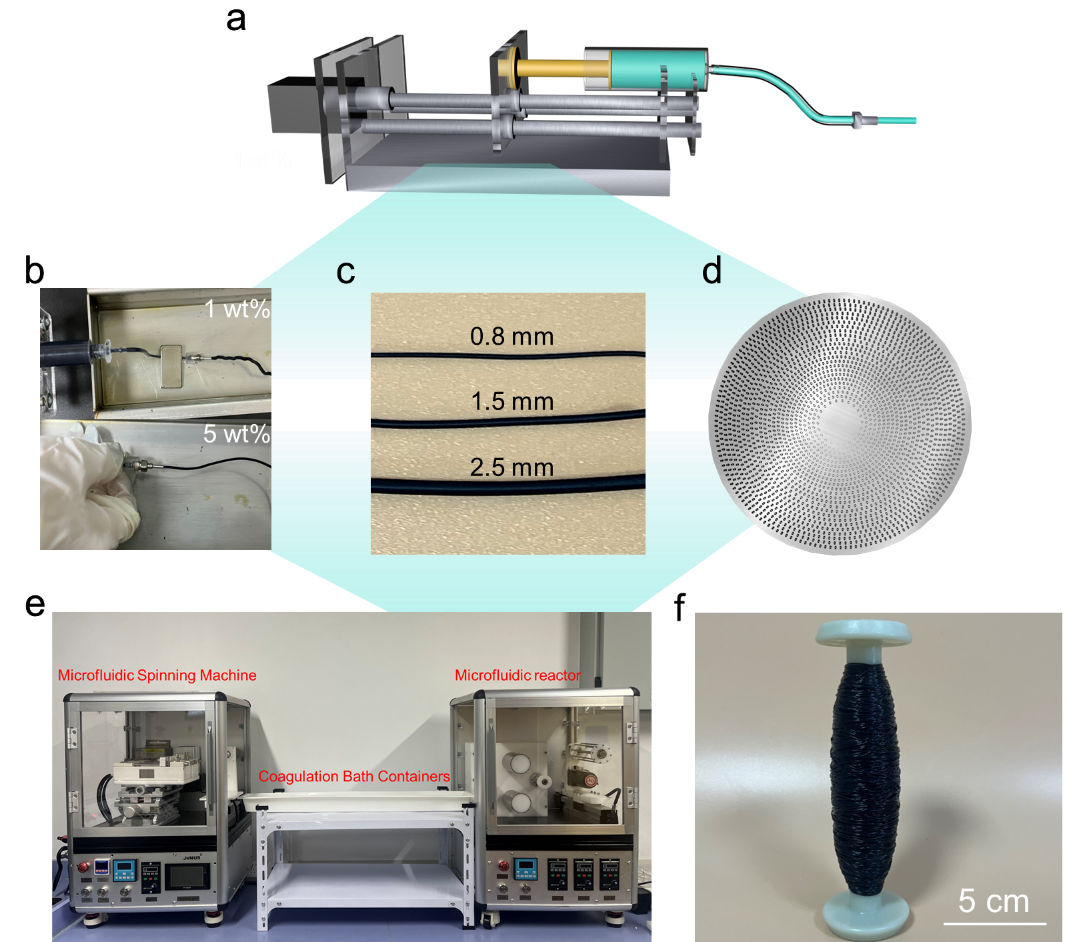


**Fig. S1 a** Schematic diagram of the setup for the preparation of hydrogel fibers. **b** Preparation of hydrogel fibers with precursor solution concentration of 1% and 5%. Here, when the solution concentration is 1 wt%, the low concentration and viscosity of the polymer result in insufficient inter-chain interactions, which broke the maintenance of fiber integrity during shear flow. Consequently, the spinning solution cannot form a continuous and stable fiber structure at the spinneret tip. Conversely, when the concentration and viscosity of the spinning solution are too high, the fibers became heterogeneous. Additionally, at high concentration, the diffusion crosslinking rate of Ca^2+^ is slowed down, impacting the gelation kinetics of the hydrogel fibers. Based on these analyses, the optimal concentration of the sodium alginate/MXene mixture was determined as 5 wt%. At this concentration, the fibers exhibit both continuity and mechanical stability, while the processability of the solution is maintained, achieving the best balance between kinetics and rheology during the formation process. **c** Hydrogel fibers with diameters of 0.8, 1.5, and 2.5 mm. **d** Multi-needle spinnerets commonly used in the wet-spinning process in industry. **e** The “propulsion-solidification-coiling collection” device. The precise control of the hydrogel fiber diameter was achieved by finely adjusting the advancement speed of the micro syringe pump and varying the needle diameter, resulting in hydrogel fibers with diameters of 0.8, 1.5, and 2.5 mm. To facilitate the subsequent adjustment of the large-scale space between the adjacent fibers, the diameter of the hydrogel fibers used in the HFCA evaporator was 0.8 mm (The optimal porosity of 10% is valid within the context of a fixed fiber diameter of 0.8 mm, and that systematic re-optimization would be required when the fiber diameter is varied), with the syringe pump’s propulsion speed maintained at 0.6 mm s^-1^. **f** Hydrogel fibers collected on a reel


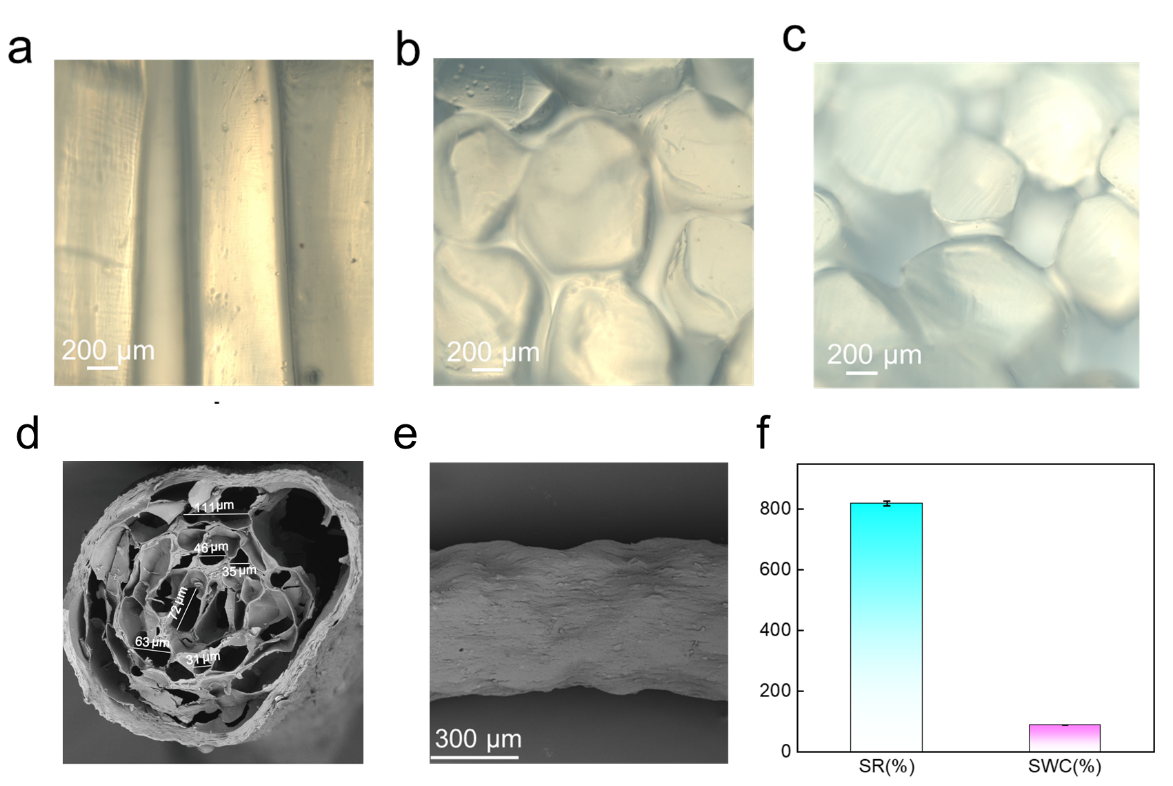


**Fig. S2 a** Optical microscope image of the vertical channel of the HFCA evaporator. **b c** Optical microscope images of large-scale spaces between fibers of HFCA-3 and HFCA-15 evaporators without MXene. **d** SEM image of the internal pores within the hydrogel fiber. **e** SEM image of HFCA surface. **f** Swelling ratio and saturated water content of HFCA


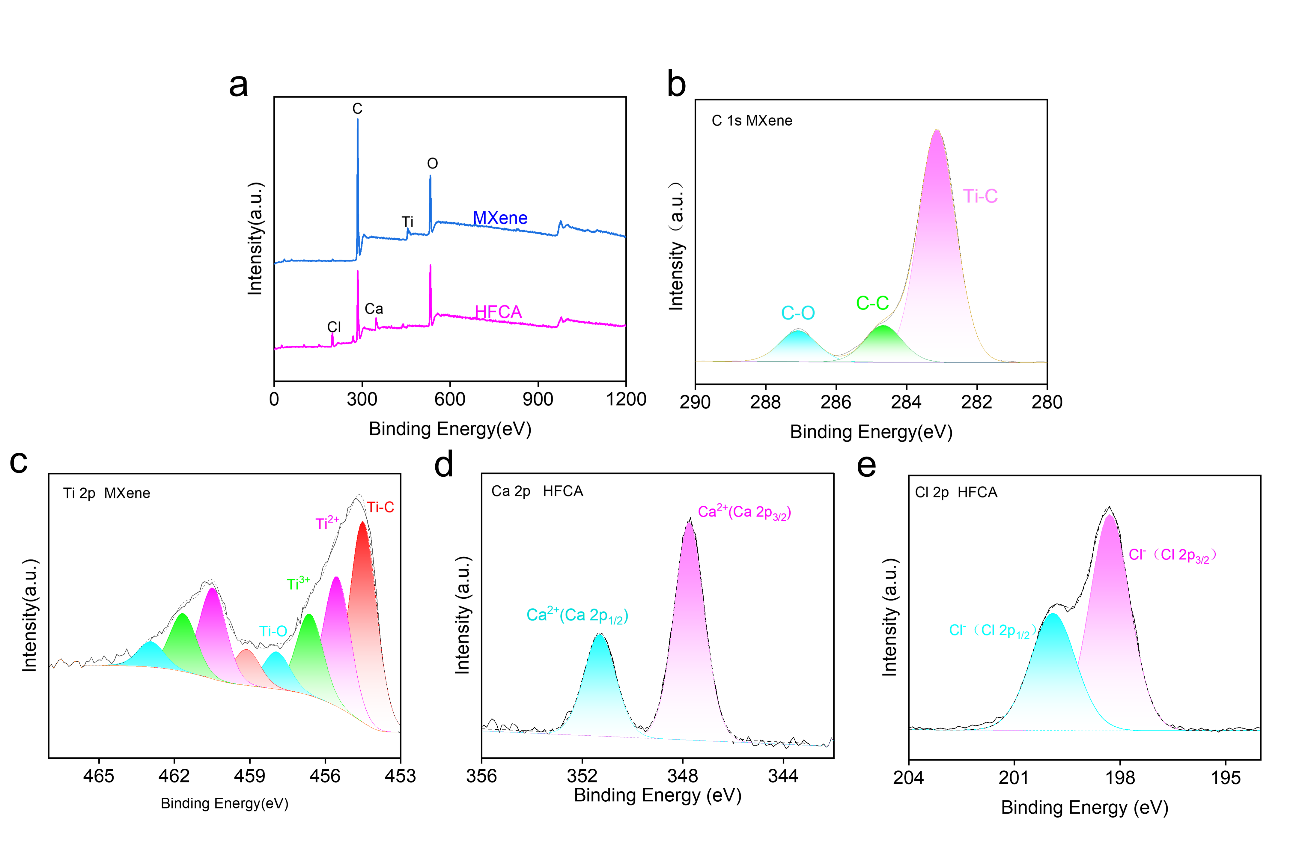


**Fig. S3 a** XPS full spectrum of MXene and HFCA. b, c High-resolution XPS spectra of C1s in the MXene. **d, e** High-resolution XPS spectra of Cl 2p, Ca 2p and O 1s, in the HFCA


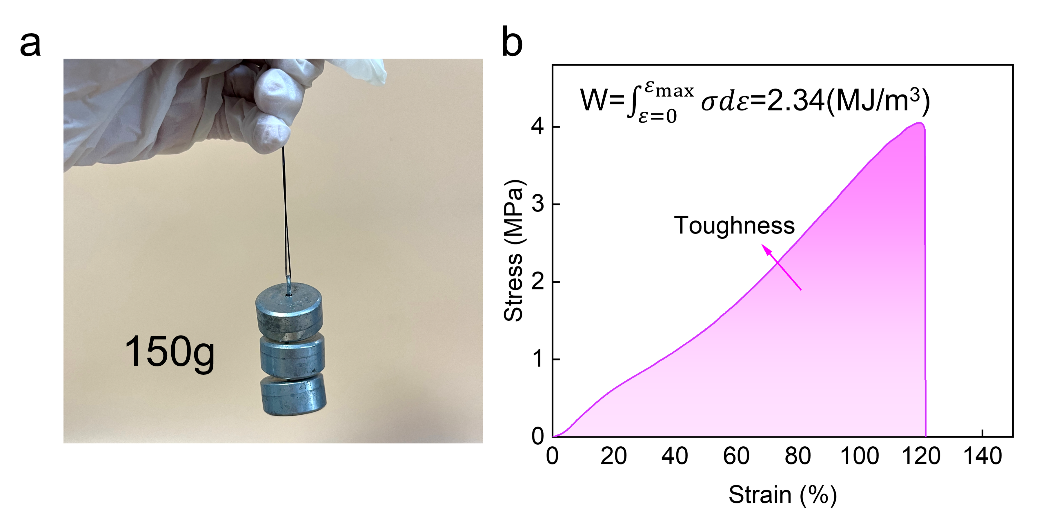


**Fig. S4 a** HFCA with diameter of 0.8 mm could lift weight of 150 g. **b** Stress-strain curves of HFCA with diameter of 0.8 mm


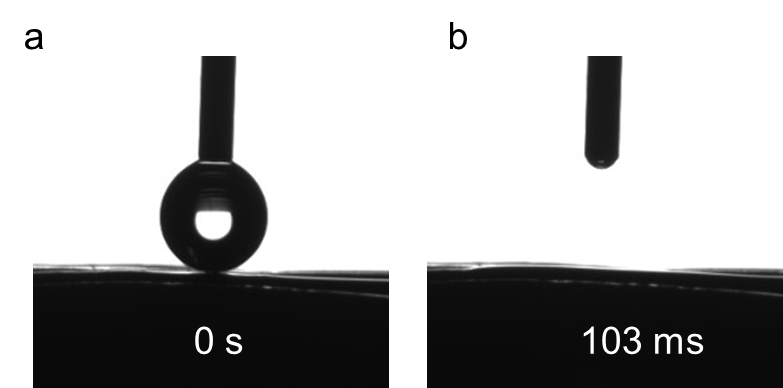


**Fig. S5** Hydrophilicity test of HFCA under contact angle meter


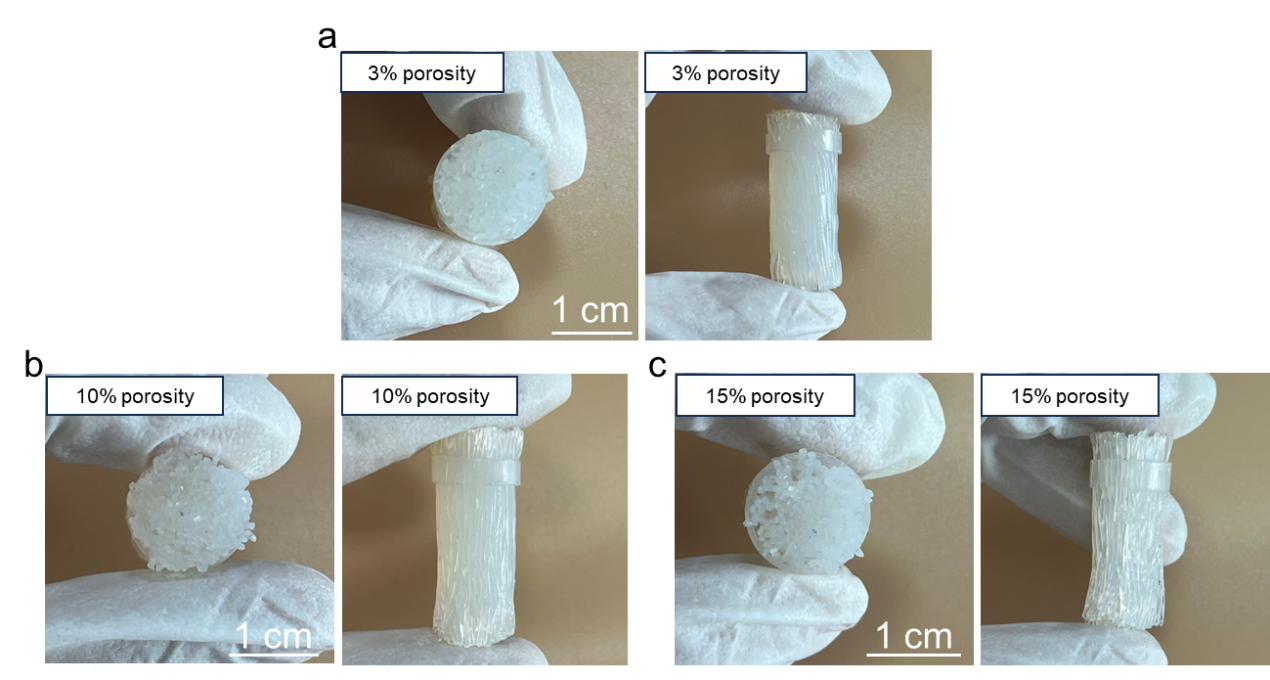


**Fig. S6** Cross-sectional images of HFCA evaporators with **a** 3%, **b** 10%, and **c** 15% porosity


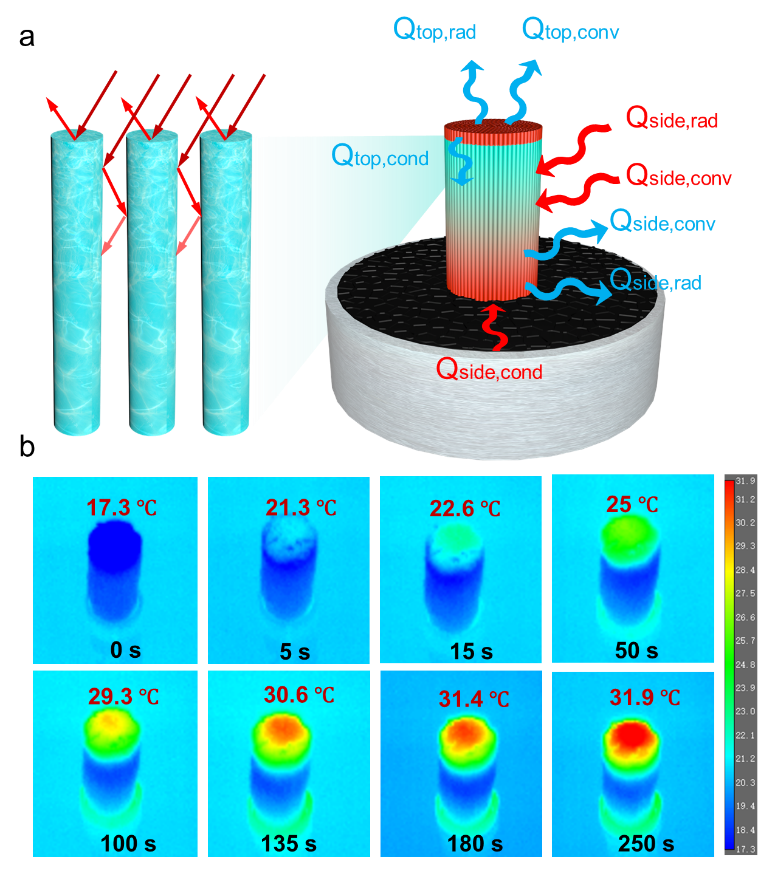


**Fig. S7 a** Light absorption of vertical channels (left). The energy obtained by HFCA evaporator from the environment based on the “heat supply/insulation model” (right). The blue arrows represent the lost heat energy. The red arrows represent the obtained energy from the environment. **b** The variation of temperatures at the top surface of the evaporator without water in the large-scale spaces under one simulated solar intensity

**
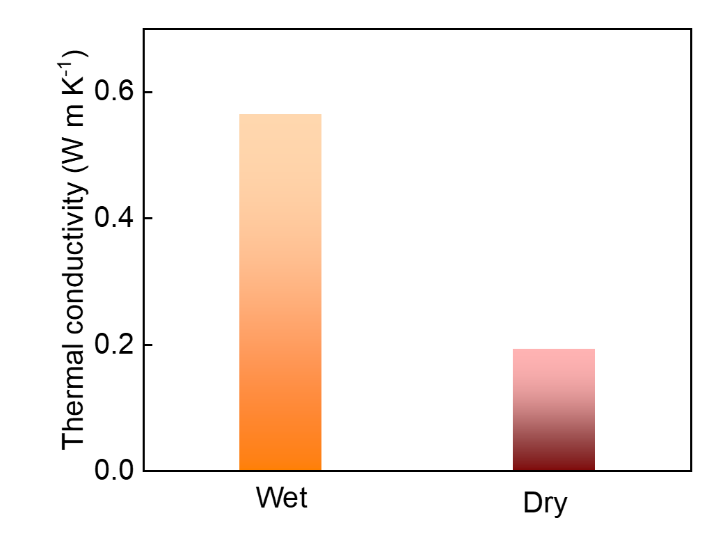
**

**Fig. S8** Longitudinal thermal conductivity of HFCA evaporator (with and without water in the large-scale spaces)


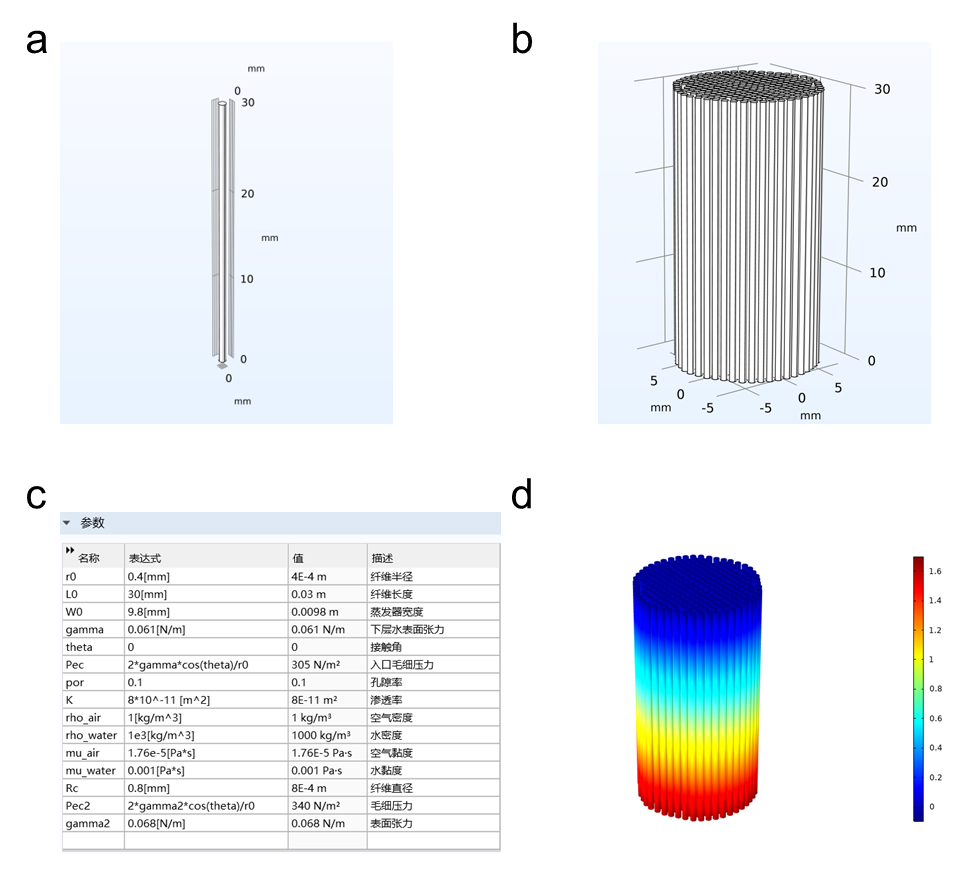


**Fig. S9** **a** 3D models of hydrogel fiber and **b** HFCA-10 evaporator in COMSOL software. **c** Parameters for COMSOL simulations. **d** Darcy velocity field simulated by COMSOL


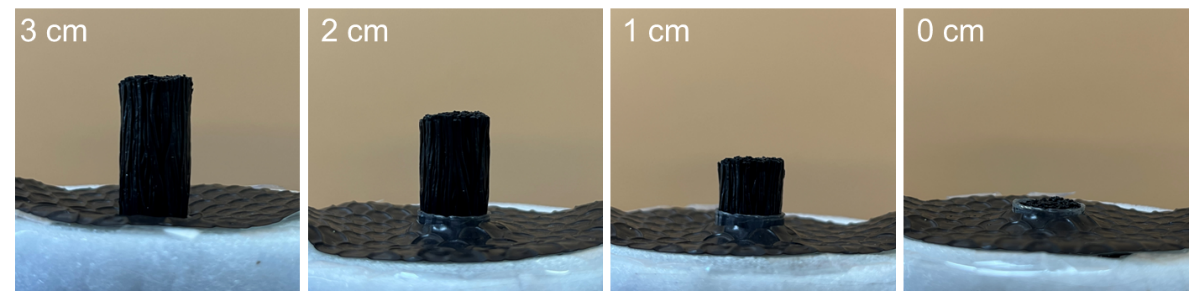


**Fig. S10** HFCA-10 evaporators at different heights

**
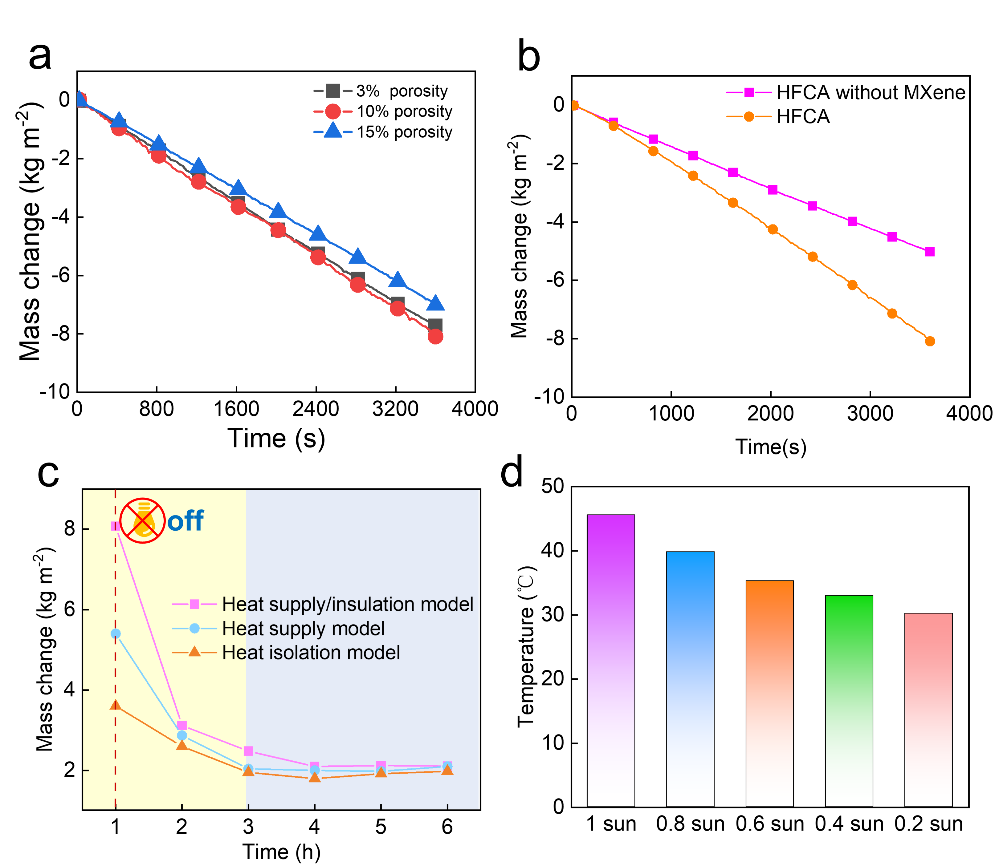
**

**Fig. S11 a** Evaporation rates of HFCA evaporators with different porosities. **b** Evaporation rates for HFCA and HFCA evaporators without MXene. **c** Evaporation rates of different models for HFCA evaporators after turning off the lights. **d** Temperature of bulk water at different solar intensities


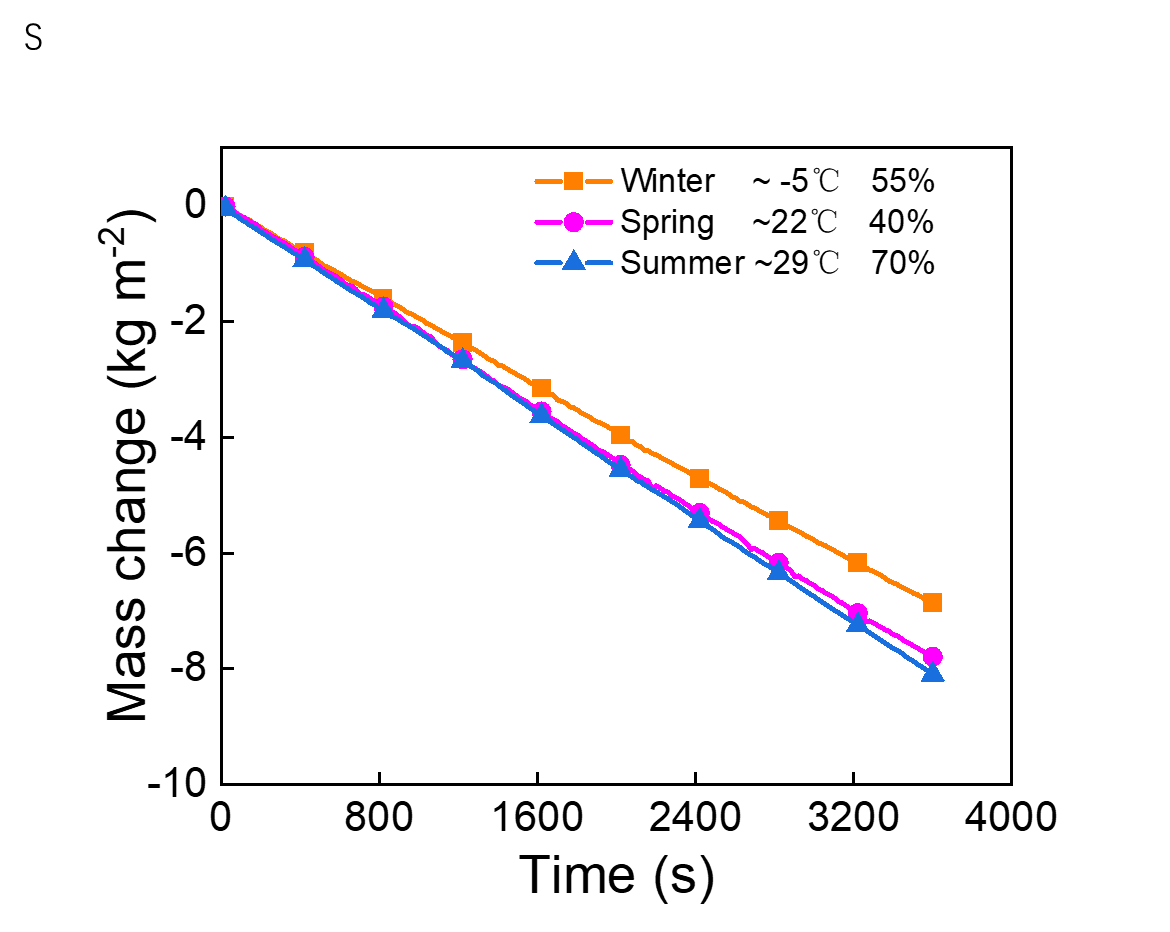


**Fig. S12** Evaporation rates of HFCA in different environments


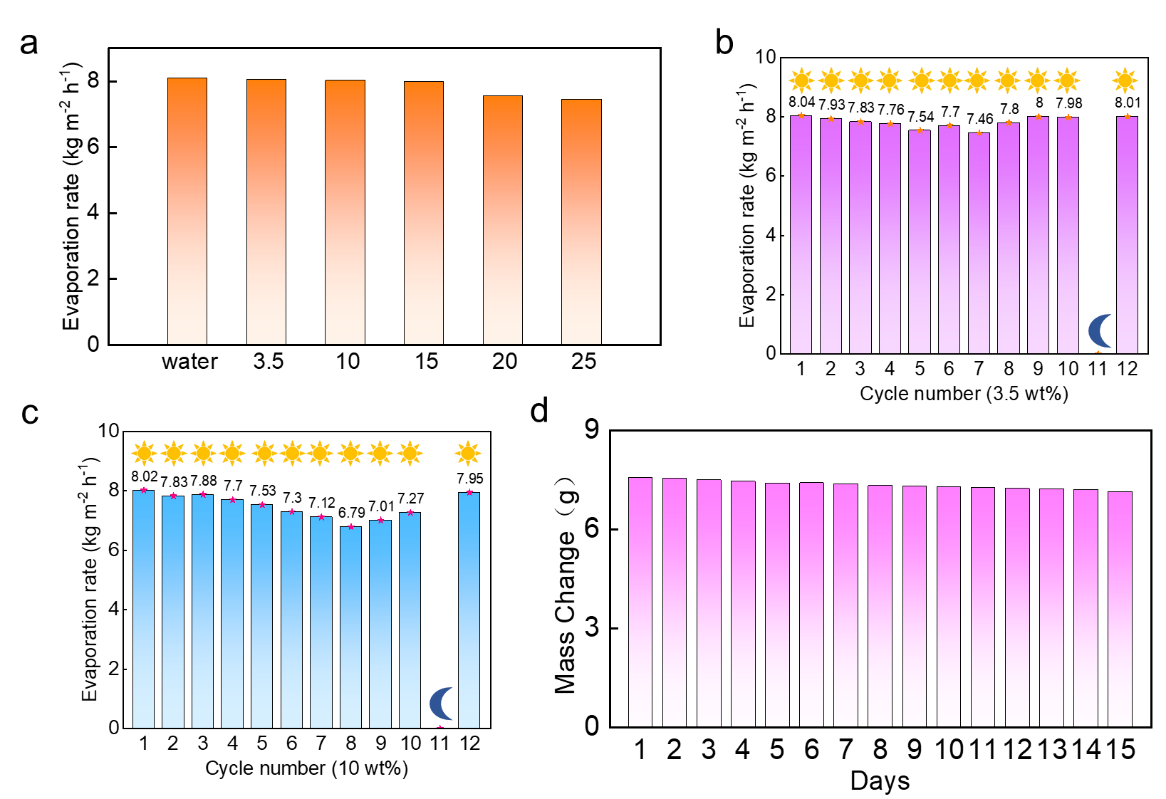


**Fig. S13 a** Evaporation rates of brines with different concentrations. **b, c** The HFCA-10 evaporator simulated diurnal evaporation rate changes at 3.5 wt% and 10 wt% brine. **d** Stability of the evaporator over a long period of time


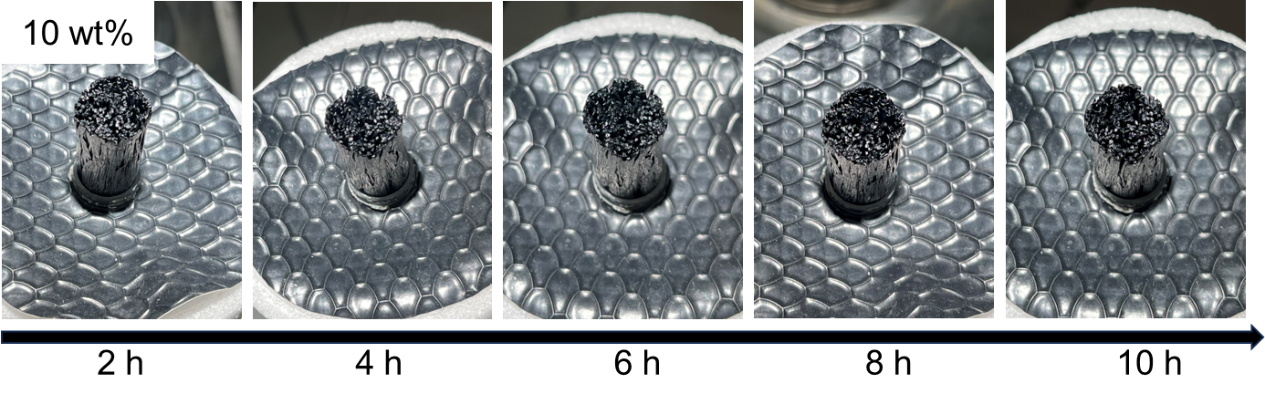


**Fig. S14** The HFCA evaporator was operated continuously for 10 h in 10 wt% brine without salt crystallization on the surface


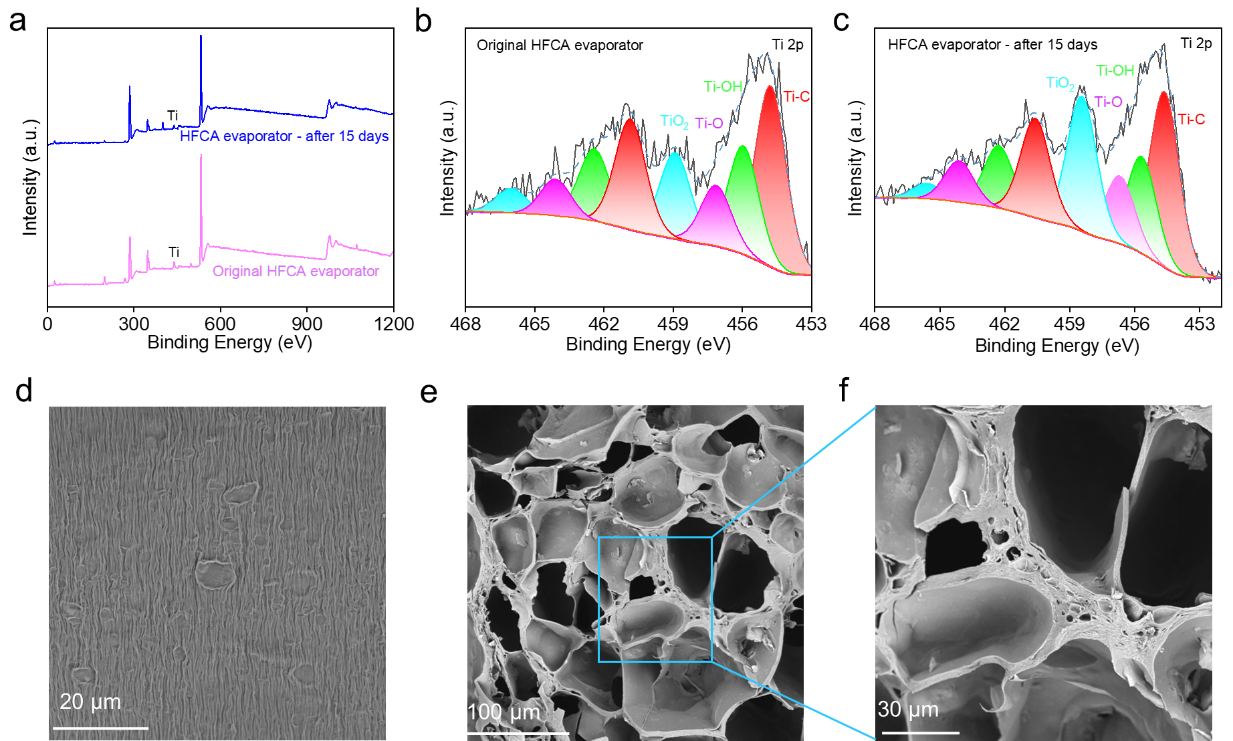


**Fig. S15 a** XPS full spectrum of original HFCA evaporator and HFCA evaporator after 15 days. **b, c** High-resolution XPS spectra of Ti 2p in the HFCA evaporator. **d** Surface of HFCA evaporator fibers after 15 days of operation. **e, f** Cross-section of fibres after 15 days of HFCA evaporator operation


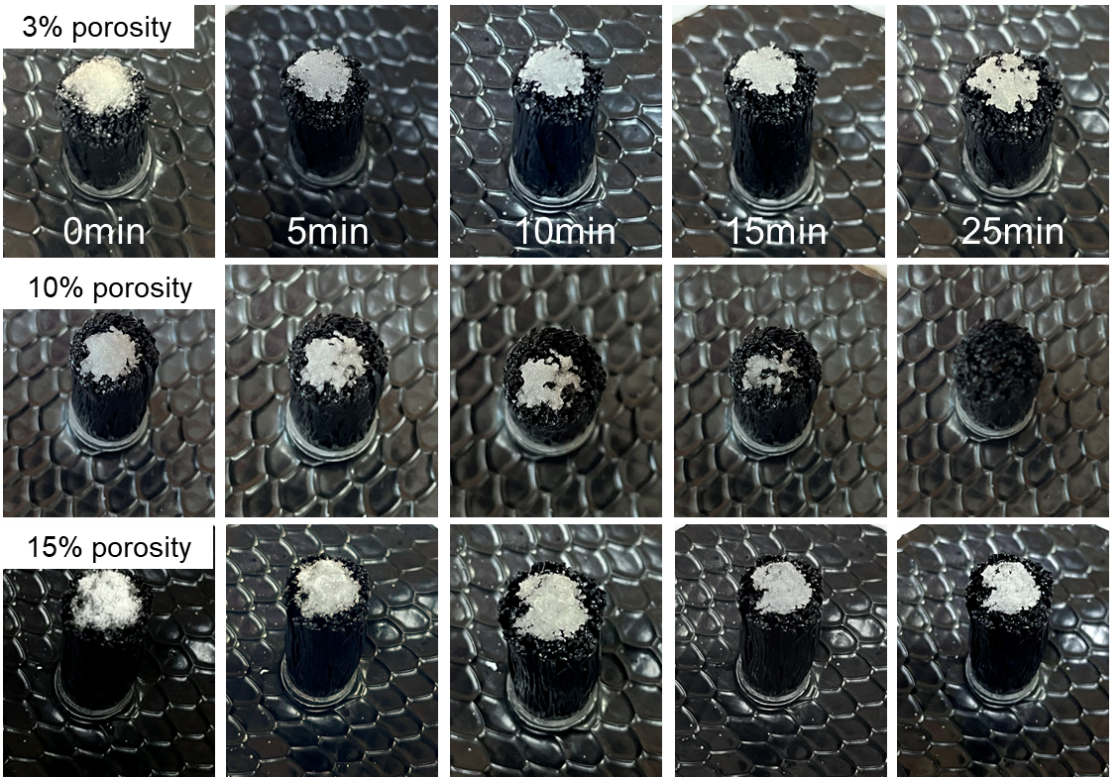


**Fig. S16** Self-cleaning dissolution of NaCl on the surface of the HFCA-3, HFCA-10, and HFCA-15 evaporators


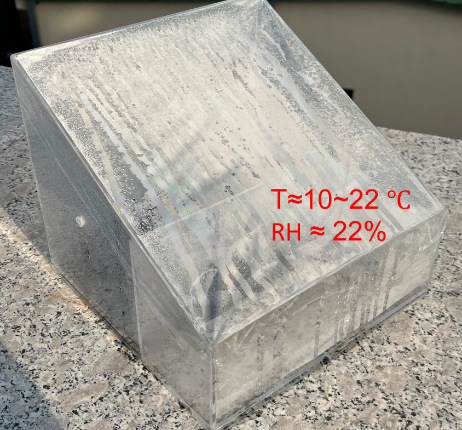


**Fig. S17** The setup photo of outdoor test (Spring)


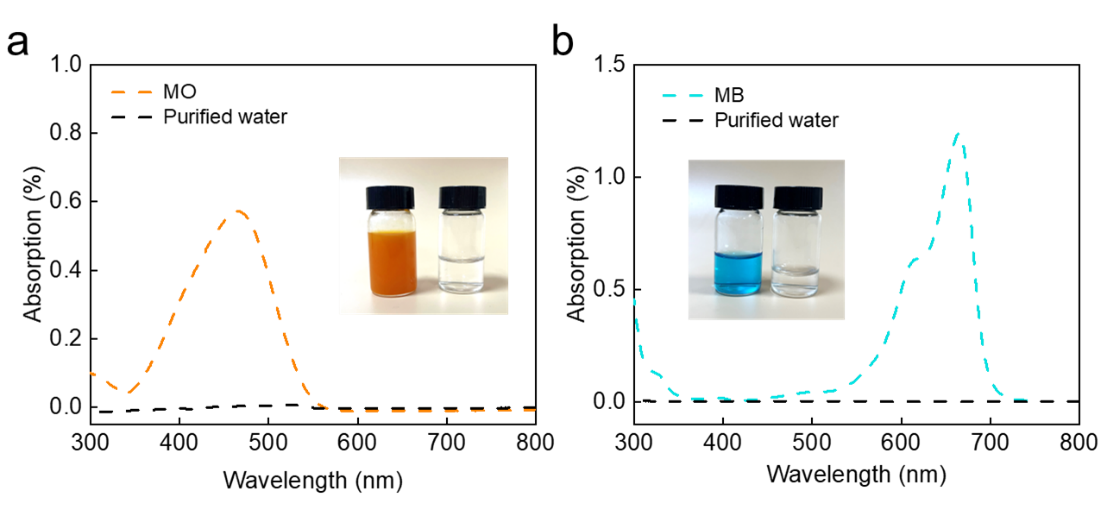


**Fig. S18** Absorption spectra of **a** methyl orange (MO) and **b** methylene blue (MB) solutions before evaporation and the corresponding purified water after evaporation


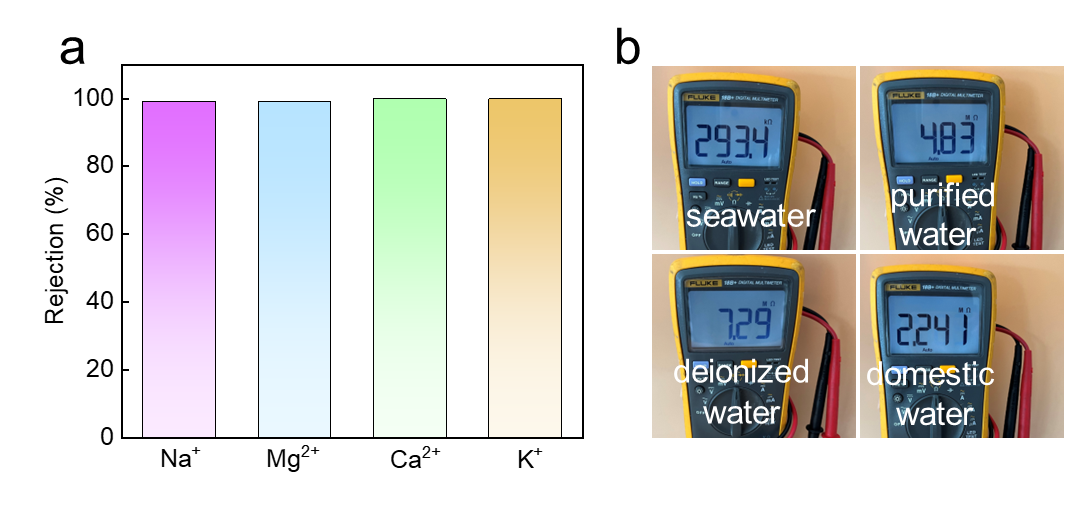


**Fig. S19 a** Rejection of four major ions (Na^+^, Mg^2+^, Ca^2+^, and K^+^) after evaporation. **b** Resistance of seawater, purified water, deionized water, and domestic water


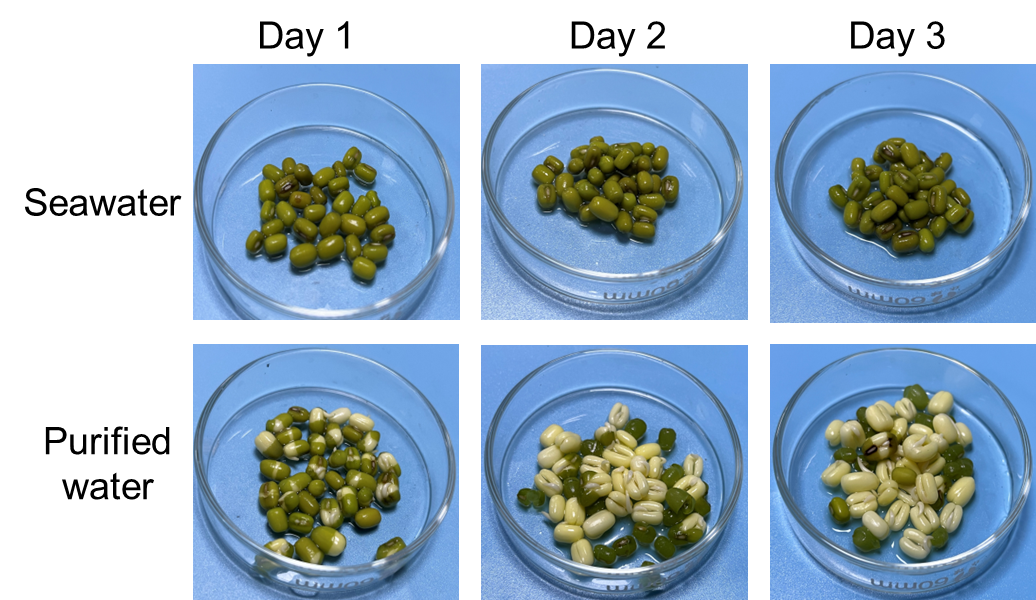


**Fig. S20** The growth and development of seeds in seawater and purified water. Seawater could drain water from plant cells due to salinity osmotic pressure, resulting in cell dehydration, while the safety and cleanliness of purified water ensured proper growth and development of seeds. This highlighted the importance of effective desalination processes in providing water suitable for agricultural and ecological applications

**
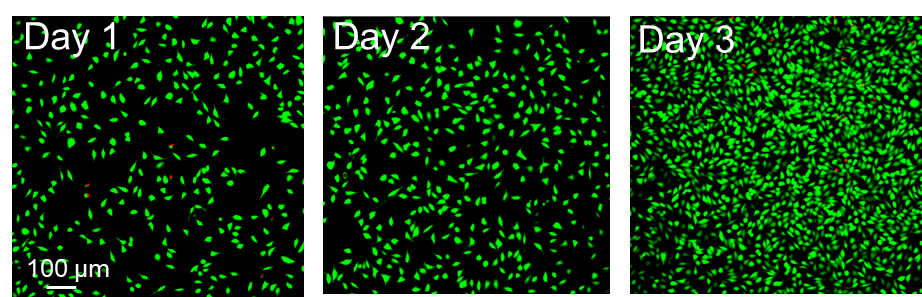
**

**Fig. S21** The images of fibroblasts cultured in double-distilled water from HFCA-10 evaporator

**Supplementary Tables**

**Table S1** Variation in evaporation rates across different evaporation models

|  | Heat isolation model  (Side Evaporation/  Total Evaporation) | Heat supply model  (Side Evaporation/  Total Evaporation) | | Heat supply/  isulation model  (Side Evaporation/  Total Evaporation) |
| --- | --- | --- | --- | --- |
| 0 cm | 1.7391 (-) | | 3.069 (-) | 4.836 (-) |
| 1cm | 2.604 (33.2%) | | 4.092 (-) | 6.324 (-) |
| 2cm | 3.255 (46.6%) | | 5.487 (68.3%) | 6.7332 (74.2%) |
| 3cm | 3.627 (52.1%) | | 5.5149 (68.5%) | 8.091 (78.5%) |

**Table S2** The evaporation rate of reported evaporators with different matrix

| Evaporator | Evaporation rate  (kg m^-2^ h^-1^) | Refs.  in the Text | Model |
| --- | --- | --- | --- |
|  | 8.09 | This work | Heat supply/  insulation model |
| **hydrogel**  **fiber evaporator** | 2.49 | [S33] | Heat isolation model |
|  | 3.80 | [S36] | Heat isolation model |
|  | 3.43 | [S37] | Heat isolation model |
|  | 4.13 | [S42] | Heat isolation model |
|  | 6.278 | [S43] | Heat isolation model |
| **hydrogel**  **evaporator** | 4.79 | [S34] | Heat isolation model |
|  | 2.41 | [S35] | Heat isolation model |
|  | 4.55 | [S38] | Heat isolation model |
|  | 4.75 | [S41] | Heat isolation model |
|  | 4.1 | [S45] | Heat supply model |
|  | 6.30 | [S46] | Heat isolation model |
|  | 3.95 | [S39] | Heat isolation model |
| **fabric evaporator** | 6.12 | [S44] | Heat isolation model |
|  | 7.14 | [S47] | Heat isolation model |
| **aerogel evaporator** | 2.28 | [S40] | Heat isolation model |

**Supplementary References**

1. N. Wu, H. Yu, M. Su, Z. Li, F. Li et al., Investigation on the structure and mechanical properties of highly tunable elastomeric silk fibroin hydrogels cross-linked by γ‑Ray radiation. ACS Appl. Bio Mater. **3**, 721-734 (2020). https://dx.doi.org/10.1021/acsabm.9b01062
2. W. Li, X. Tian, X. Li, S. Han, C. Li et al., Ultrahigh solar steam generation rate of a vertically aligned reduced graphene oxide foam realized by dynamic compression. J. Mater. Chem. A **9**(26), 14859-14867 (2021). <https://doi.org/10.1039/d1ta03014k>
